# Everyday State Attachment: Dynamic Features and Role of Trait Attachment

**DOI:** 10.1111/jopy.12975

**Published:** 2024-10-17

**Authors:** Jaakko Tammilehto, Aleksandra Kaurin, Guy Bosmans, Peter Kuppens, Marjo Flykt, Mervi Vänskä, Kirsi Peltonen, Jallu Lindblom

**Affiliations:** ^1^ Faculty of Social Sciences/Psychology Tampere University Tampere Finland; ^2^ Department of Psychology and Logopedics, Faculty of Medicine University of Helsinki Helsinki Finland; ^3^ Clinical Child and Adolescent Psychology and Psychotherapy University of Wuppertal Wuppertal Germany; ^4^ Faculty of Psychology and Educational Sciences KU Leuven Leuven Belgium; ^5^ INVEST Research Flagship Center University of Turku Turku Finland

**Keywords:** attachment system, attachment theory, dynamic structural equation model, ecological momentary assessment, state attachment

## Abstract

**Objective:**

Attachment research has traditionally focused on traits, enhancing our understanding of attachment‐related individual differences. However, to chart the dynamic properties of the attachment system, more research is needed on the within‐person fluctuation of attachment states. In this ecological momentary assessment (EMA) study, we examined (a) the associations between the baseline, variability, and inertia of each state attachment dimension (security, avoidance, and anxiety) and (b) how trait attachment (anxiety and avoidance) predicts these dynamic features.

**Method:**

In two adult samples (*N*s = 122 and 127), trait attachment dimensions were first assessed using Experiences in Close Relationships–Revised. Then, attachment states were assessed seven or ten times a day over 1 week (4629 and 5322 successful EMA observations).

**Results:**

For state security, individuals with high baseline exhibited lower variability. In contrast, for state avoidance, those with high baseline showed higher variability. Both trait attachment anxiety and avoidance predicted lower baseline and higher variability of state security. Moreover, both trait dimensions predicted higher baselines of the corresponding states.

**Conclusions:**

Our findings provide insights into the real‐time regulatory dynamics of the attachment system and their interconnection with trait attachment, underscoring the importance of baseline and variability in understanding how attachment manifests in everyday life.

## 1 Introduction

The attachment system, a motivational mechanism activated by perceived threats, drives individuals to seek proximity and protection from their attachment figures (Bowlby 1982). This regulatory function has been demonstrated in both children (Ainsworth et al. 1978) and adults (Mikulincer, Gillath, and Shaver 2002). Most research has focused on examining attachment from the trait perspective, stressing the importance of relatively stable beliefs and expectations about oneself and others (Mikulincer and Shaver 2023). These dominant representations form the basis of individuals' trait attachment, shaping the behaviors that follow from the activation of the attachment system. However, a more nuanced understanding of the attachment system and its functioning could be achieved by focusing on its moment‐to‐moment dynamics. State attachment, which refers to momentarily activated attachment representations, allows context‐sensitive attachment responses (Bosmans et al. 2020; Gillath et al. 2009). As such, it has a critical role in fostering socioemotional adaptation to a variety of daily situations. Recent advancements in ecological momentary assessment (EMA) research have provided access to real‐time state attachment fluctuations (Kaurin, Pilkonis, and Wright 2022; Tammilehto et al. 2022). By assessing state attachment multiple times per day over several days, EMA allows researchers to explore the patterns, boundaries, and regularities of people's attachment systems. Yet, our understanding of such dynamic features remains limited. In this study, we first use EMA to gain insights into the dynamic features of state attachment and their interrelations and, second, to examine how trait attachment is linked to the dynamic features.

### 1.1 Prototypical Attachment Representations: Trait Attachment

Trait attachment can be conceptualized as a person's dominant, prototypical attachment beliefs and expectations (Mikulincer and Shaver 2023). These representations develop and are strengthened through recurrent experiences with one's attachment figures (Arriaga et al. 2018; Bosmans et al. 2020). Psychometric research using self‐reports shows that individual differences in trait attachment are characterized by a two‐dimensional space of anxiety and avoidance, with low levels of both indicating trait attachment security (Raby, Fraley, and Roisman 2021).

The anxiety dimension reflects uncertainty about others' availability and concerns about one's own ability to cope with threats (Fraley, Waller, and Brennan 2000). People with high trait anxiety use strategies that hyperactivate the attachment system, such as heightened attentiveness for rejection and threat cues, along with excessive reassurance‐seeking (Brennan and Carnelley 1999; Mikulincer and Shaver 2023; Shaver, Schachner, and Mikulincer 2005). The avoidance dimension signifies a rigid distrust in others' availability and concerns about expressing emotional closeness (Fraley, Waller, and Brennan 2000). People with high trait avoidance use strategies that deactivate the attachment system, such as minimization of emotions and avoidance of threats (Mikulincer and Shaver 2023; Tammilehto et al. 2023).

### 1.2 Short‐Term Dynamics of Attachment Representations: State Attachment

Although people have dominant attachment representations, they also harbor a variety of other attachment representations, shaped by a mix of positive and negative experiences throughout their lives (Arriaga et al. 2018; Baldwin et al. 1996). Accordingly, even subtle cues indicating an attachment figure's unavailability can elicit moments of insecurity, whereas positive thoughts about others' availability may induce a sense of security (Bosmans, Bowles, et al. 2014; Gillath et al. 2009). Thus, everyone can experience both moments of security and insecurity in response to varying situational cues (Arriaga et al. 2018). These fluctuating attachment states play a critical role in guiding an individual's specific behaviors in a context‐sensitive manner (Bosmans et al. 2020; Gillath et al. 2009).

Psychometric research delineates the dimensions of state attachment security, avoidance, and anxiety (Gillath et al. 2009; Tammilehto et al. 2022). State security is characterized by the sense of being loved and cared for, embodying a set goal that the attachment system strives to attain and maintain whenever possible. Different from the trait level, security is an independent dimension at the state level, distinct from state anxiety and avoidance. In turn, state avoidance is characterized by a fear of losing independence, along with attempts to deactivate the attachment system to avoid expected painful rejection from others. Finally, state anxiety is characterized by a strong urge for affection and care, along with the hyperactivation of the attachment system to enhance actual or symbolic proximity to the attachment figure (Gillath et al. 2009; Tammilehto et al. 2022).

A growing body of research has examined state attachment dynamics in daily life. Diary studies assessing state attachment once daily over multiple days indicate that receiving interpersonal support covaries with heightened security and reduced anxiety and avoidance (Bosmans, Van de Walle, et al. 2014; Haak, Keller, and DeWall 2017; Verhees et al. 2023; Zhang 2009). Moreover, two EMA studies show significant moment‐to‐moment variations in state attachment security, avoidance, and anxiety that may both influence and be influenced by emotion regulation and interpersonal processes (Kaurin, Pilkonis, and Wright 2022; Tammilehto et al. 2022). In sum, research indicates that people undergo substantial fluctuations in state attachment, which show covariance with theoretically relevant processes.

### 1.3 Basic Dynamics Features of State Attachment: Baseline, Variability, and Inertia

**FIGURE 1:**
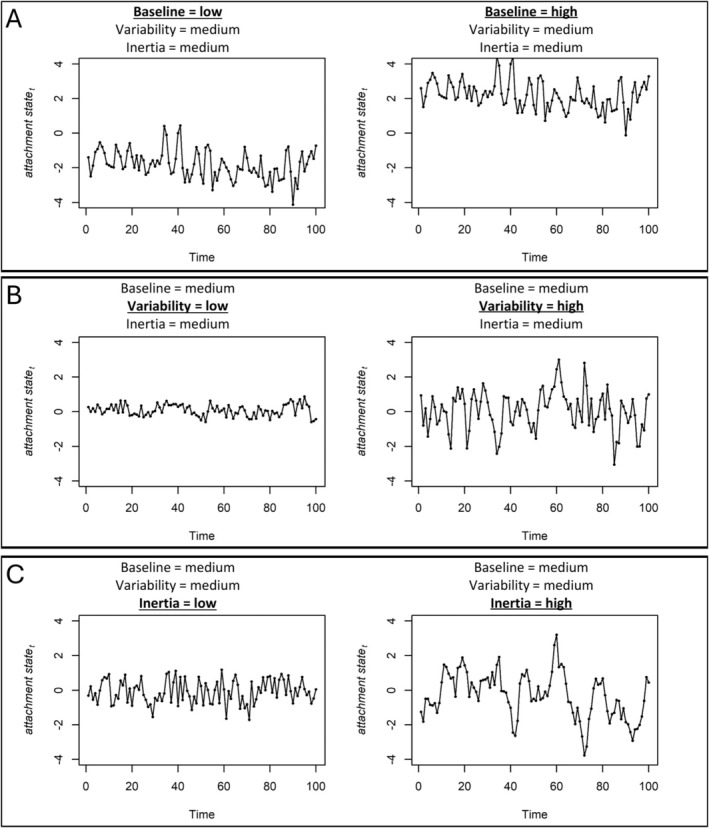
Illustration of basic dynamic features of state attachment: simulations for a person with high and low levels of baseline (A), variability (B), and inertia (C) while keeping other features constant. Panels A, B, and C illustrate that each dynamic feature captures an independent aspect of state attachment fluctuations. Baseline (A) captures the long‐term average around which an individual's attachment state fluctuates over time. Variability (B) reflects the moment‐to‐moment fluctuations in the attachment state in response to unobserved external and internal factors. Inertia (C) represents the predictable influences on how quickly (low inertia) or slowly (high inertia) an individual returns to their baseline after experiencing perturbations. As shown in Panels A, B, and C, individuals with the same levels of other dynamic features can exhibit notable differences in their baseline, variability, and inertia. Thus, each dynamic feature represents an independent component that contributes to the flow of attachment states in everyday life. R script for the simulations is available at https://osf.io/qke5m/.

Despite the progress in state attachment research, our understanding of dynamic features that capture the temporal nature of attachment representations remains limited. A thorough investigation of these features can increase our understanding of attachment phenomena and their role in well‐being. The basic dynamic features of state attachment encompass baseline, variability, and inertia, all stemming from the field of affect dynamics, which analyzes emotional patterns across time (Kuppens, Oravecz, and Tuerlinckx 2010). Figure 1 shows people with high and low levels of baseline, variability, and inertia, while other dynamic features are kept constant.

The concept of baseline (Figure 1A) refers to a person's equilibrium or the average level of each state attachment dimension over an extended period (Hamaker et al. 2018; Kuppens, Oravecz, and Tuerlinckx 2010). This feature closely aligns with trait attachment conceptually, as the average is expected to mitigate transient situational fluctuations. Yet, baseline is more sensitive to state‐like variations over time. Thus, the baselines of each state attachment dimension reflect a ‘home base’ of the attachment system around which attachment responses revolve (Dugan et al. 2024).

Variability (Figure 1B), in the attachment framework, captures the short‐term, momentary oscillations in each state attachment dimension away from the baseline (Dugan et al. 2024). Such fluctuations reflect the individual's reactivity and adaptability to immediate environmental or internal changes (Hamaker et al. 2018; Kuppens, Oravecz, and Tuerlinckx 2010). High variability indicates heightened sensitivity of the attachment system, enabling rapid reactive responses (Dugan et al. 2024). Thus, this feature depicts the dynamic essence of the attachment system as being responsive to the continuous flow of interpersonal interactions and personal experiences (Arriaga et al. 2018; Bosmans et al. 2020).

Finally, inertia (Figure 1C) has recently garnered attention in attachment research (Verhees et al. 2023). It quantifies the temporal stability of each attachment state, indicating how attachment states carry over from one moment to the next (Kuppens, Oravecz, and Tuerlinckx 2010). This temporal stability of attachment states differs from the more widely studied prototype‐like stability in trait attachment research. Whereas prototype‐like stability pertains to enduring characteristics that define time‐invariant differences over long periods (Fraley et al. 2011), inertia focuses on the short‐term continuity of specific states, capturing how current attachment states influence immediate future states (Kuppens, Oravecz, and Tuerlinckx 2010). Thus, inertia reflects how promptly or slowly the attachment states revert to their baselines, indicating how long the attachment system lingers after facing positive or negative shocks. Moreover, while it might be tempting to interpret inertia as the opposite of variability, they reflect independent features. Inertia captures how current attachment states influence immediate future states, while variability reflects all unobserved external and internal influences on attachment states. As shown in Figure 1B,C, people with the same level of inertia can exhibit different levels of variability, and vice versa.

Exploring the connections between baseline, variability, and inertia within each state attachment dimension using EMA is a new area of research. This approach has the potential to provide valuable insights into the organization of the attachment system and individual differences. Despite the novelty of this research, some hypotheses can be derived from attachment theory and related diary studies.

Previous research suggests that individuals with high baseline state security tend to process interpersonal and threatening situations in a positively biased manner, aligning with their secure beliefs and expectations (Bosmans et al. 2020; Dykas and Cassidy 2011; Mikulincer and Shaver 2023). As a result, situational factors may be less likely to cause rapid deviations in state security for these individuals, leading to lower variability and greater inertia in this dimension (Verhees 2019). This hypothesis is supported by two daily diary studies, which found that children with high baseline state security also exhibited reduced variability and higher inertia in their security (Bosmans, Van de Walle, et al. 2014; Verhees 2019). However, these findings might partly reflect statistical ceiling effects, which limit the variance at the upper end of the measurement scale.

Compared to security, diary research into the dynamic features of state attachment avoidance and anxiety is scarcer. One adult diary study found that high baseline state avoidance correlated with greater state avoidance variability, and, similarly, high baseline state anxiety correlated with greater state anxiety variability (Haak, Keller, and DeWall 2017). These preliminary findings suggest that the dynamic features of avoidance and anxiety might differ from those of security, reflecting the distinct functional purposes of secure and insecure states.

Finally, differences in trait attachment can be hypothesized to influence the dynamic features of state attachment, as dominant attachment representations shape attachment‐related information processing (Dykas and Cassidy 2011). Individuals with low trait attachment anxiety and avoidance may exhibit higher baseline, lower variability, and greater inertia in state security, reflecting the predominance of security‐laden appraisals (Bosmans et al. 2020; Verhees 2019). Supporting this hypothesis, a diary study in children found that low trait anxiety and avoidance predicted lower variability in state security, and low trait avoidance predicted higher baseline state security (Bosmans, Van de Walle, et al. 2014). Additionally, two diary studies in adults showed that individuals with high trait attachment anxiety and avoidance exhibited higher baselines of the corresponding attachment states (Haak, Keller, and DeWall 2017; Sibley, Fischer, and Liu 2005). These findings align with the conceptual correspondence between trait attachment and state attachment baselines, further substantiating the role of trait attachment in the dynamic features of state attachment.

However, previous research is subject to several critical limitations. First, there is a notable absence of studies on the inertia of state attachment avoidance and anxiety. Second, studies on state attachment variability have focused solely on net variability (also known as total variance). Alarmingly, net variability is statistically dependent on the amount of inertia (Jongerling, Laurenceau, and Hamaker 2015). In recent years, sophisticated techniques like dynamic structural equation modeling (DSEM) have been developed to decompose the innovation variance, an indicator of variability that is independent of inertia (Asparouhov, Hamaker, and Muthén 2018). DSEM can also account for ceiling and floor effects (Muthén, Asparouhov, and Shiffman 2024), which have hindered interpretations of some previous state attachment studies. Finally, the diary designs used in previous research on dynamic features capture only day‐to‐day fluctuations, limiting insights into moment‐to‐moment attachment dynamics. These real‐time dynamics can be better captured using EMA. In the current study, we aim to address all these limitations in the extant state attachment research.

### 1.4 Current Study

In this study, we examined the dynamic features of state attachment and the associations of trait attachment with these features. To address limitations in previous research, we utilized DSEM to analyze three dynamic features—baseline, variability, and inertia—within each state attachment dimension across two EMA samples. Our ultimate goal was to enhance our understanding of how the attachment system operates dynamically in everyday life.

Our first aim was to explore the associations between baseline, variability, and inertia within each dimension of state attachment, focusing on security, avoidance, and anxiety. According to our first preregistered hypothesis, supported by previous diary research, high baseline state security would be associated with lower variability and greater inertia in state security. We also explored similar associations within state anxiety and avoidance dimensions. However, due to the limited existing research and the absence of strong theoretical predictions, we did not formulate predefined hypotheses for these dimensions. Thus, the aim of these preregistered exploratory analyses was to identify consistent patterns that could provide insights into state attachment dynamics and generate new hypotheses.

Our second aim was to investigate the associations of trait attachment with the dynamic features of state attachment. Our second preregistered hypothesis, supported by previous diary research, proposed that individuals with low trait attachment anxiety and avoidance would demonstrate higher baseline, lower variability, and greater inertia in state security. Additionally, our third and fourth preregistered hypotheses suggested that high trait attachment avoidance would predict higher baseline state avoidance, and high trait attachment anxiety would predict higher baseline state anxiety. Beyond these predefined hypotheses, we conducted preregistered exploratory analyses to explore all potential associations between trait attachment dimensions and the dynamic features of each state attachment dimension. Again, this exploratory approach aimed to uncover insights and generate hypotheses on state attachment dynamics.

## 2 Methods

### 2.1 Participants and Procedure

We utilized two distinct EMA adult samples: Sample I from the Daily Emotions research project (Tammilehto et al. 2022) and Sample II from the Miracles of Development research project (https://projects.tuni.fi/kehi/). For an in‐depth description of both samples and their demographics, see Tammilehto et al. (2023). We preregistered our hypotheses and analysis plan before analyzing the data. We conducted all preregistered analyses without deviations.^1^ In addition, we conducted several sensitivity analyses to assess the robustness of our findings.

Sample I for the Daily Emotions project initially recruited 125 participants, mostly students, meeting the criteria of (a) aged over 18 years, (b) ability to use a smartphone, and (c) proficiency in Finnish. Participants were recruited through Tampere University email lists and paper flyers distributed across campus areas. Thus, the number of people who were exposed to the recruitment efforts but never contacted us was unknown. The project received ethical approval from The Ethics Committee for Humanities of the Tampere Region. Data collection consisted of two phases: an online questionnaire followed by an EMA phase approximately 2 weeks later. During the EMA phase, participants received short smartphone questionnaires seven times daily for a week. Questionnaire sending times were randomly allocated between 10:00 AM and 10:00 PM in seven 1‐h and 43‐min blocks. For one participant, the data from the first questionnaire phase were missing, and due to a technical error, two participants were assigned the same EMA identity number. These individuals were excluded from the analysis. The final sample comprised 122 participants (*M*_age_ = 26.43 years, *SD* = 8.33, range: 19–52; 88.5% women), with 77.4% compliance and 4629 EMA observations. Of the participants, 53.3% were university students, 40.2% were open university students, 4.1% were other students, and 2.5% were non‐students.

Sample II, part of the Miracles of Development project, involved young adults aged 20–22 years. The Miracles of Development project is an ongoing longitudinal study that has followed Finnish families from the second trimester of pregnancy to the child's early adulthood. All data collections have received ethical approval from the ethical boards of Helsinki University Central Hospital. The inclusion criteria for the EMA subsample included (a) no severe developmental disorders, (b) availability of address information, and (c) no expressed desire to discontinue participation. Of 710 young adults contacted via mail, 130 participated. Attrition was unrelated to various early sociodemographic and family factors reported in Tammilehto et al. (2023). Data collection comprised an online questionnaire phase and a following EMA phase about a couple of days later. The EMA phase involved short smartphone questionnaires ten times daily for a week, sent randomly between 8:00 AM and 10:00 PM in ten 1‐h and 24‐min blocks. Two participants provided fewer than three EMA responses (less than 3%), and one did not participate in any phase of the study. These individuals were excluded from the analysis. The final sample consisted of 127 participants (*M*_age_ = 20.98, *SD* = 0.45, range: 20–22; 66.9% women), with 59.9% compliance and 5322 EMA observations. Of the participants, 2.4% had the highest education level of the undergraduate degree, 84.3% matriculation examination, 9.4% vocational education and training, and 3.9% comprehensive school.

### 2.2 Measures

#### 2.2.1 Trait Attachment

Trait attachment in both samples was measured using the *Experiences in Close Relationships*–*Revised* (Fraley, Waller, and Brennan 2000). The participants reported their trait attachment anxiety (18 items; e.g., “I worry a lot about my relationships”) and avoidance (18 items; e.g., “I am nervous when partners get too close to me”) using a 7‐point Likert scale (1 = *strongly disagree* to 7 = *strongly agree*). Cronbach's alphas for trait attachment anxiety were 0.92 in Sample I and 0.93 in Sample II, and for avoidance, they were 0.91 in both samples.

#### 2.2.2 State Attachment

In the EMA phase of both samples, state attachment was measured using items from the *State Adult Attachment Measure* (SAAM; Gillath et al. 2009). The SAAM is currently the only standard measure to assess adult state attachment. Its construct and predictive validity have been supported in several studies (Bosmans, Bowles, et al. 2014; Gillath et al. 2009; Kaurin, Pilkonis, and Wright 2022). Original SAAM consists of 21 items that use a 7‐point Likert scale (1 = *strongly disagree* to 7 = *strongly agree*) to assess people's state attachment at the current moment. Regarding Sample I, to decrease participant reporting burden in the EMA, we selected six items to assess state attachment security (“I feel loved”; “I feel like I have someone to rely on”), avoidance (“If someone tried to get close to me, I would try to keep my distance”; “The idea of being emotionally close to someone makes me nervous”), and anxiety (“I feel a strong need to be unconditionally loved right now”; “I want to share my feelings with someone”). These items were selected based on their high factor loadings in the original validation study (Gillath et al. 2009) and the absence of significant content overlap between them. In Sample II, the same items were used except for one; “I want to share my feelings with someone” was replaced with “I want to talk with someone who cares for me about things that are worrying me”. This change was made because anxiety showed low reliability at the within‐person level in Sample I (see below).

In both samples, we assessed the fit of the SAAM model using multilevel confirmatory factor analyses with random intercepts. These results are reported in Supporting Information 1 (Tables S1A and S1B).^2^ In the interpretation, we followed the common benchmarks, including CFI > 0.950, RMSEA < 0.060, and SRMR < 0.080 (Hu and Bentler 1999). In Sample I, the model with the three state attachment dimensions (i.e., security, avoidance, and anxiety) at within‐ and between‐person level (Figure S1) showed adequate fit apart from between‐person SRMR, *χ* ^2^ [18] = 177.77, *p* < 0.001, CFI = 0.949, RMSEA = 0.057, SRMR_within/between_ = 0.056/0.109, *χ* ^2^/df = 9.88. In Sample II, the model with the three state attachment dimensions showed adequate fit, *χ* ^2^ [19] = 46.20, *p* < 0.001, CFI = 0.990, RMSEA = 0.020, SRMR_within/between_ = 0.016/0.069, *χ* ^2^/df = 2.43. In Sample I, omega coefficients for state attachment security, avoidance, and anxiety at the within‐person level were 0.71, 0.72, and 0.47, respectively. At the between‐person level, they were 0.92, 0.97, and 0.70, respectively. In Sample II, omega coefficients for state attachment security, avoidance, and anxiety at the within‐person level were 0.63, 0.68, and 0.41, respectively. At the between‐person level, they were 0.97, 0.98, and 0.83, respectively. Thus, while between‐person reliabilities were adequate in both samples, state attachment anxiety consistently showed lower reliabilities at the within‐person level. Nonetheless, all within‐person reliabilities conformed to the benchmarks outlined in the EMA literature for acceptable levels (Nezlek 2017).

#### 2.2.3 Covariates

When testing the associations of trait attachment, we controlled for participants' relationship status (0 = *single*, 1 = *in a romantic relationship*) and the proportion of EMAs where they reported being alone. This approach enabled us to consider the potential confounding effects of romantic relationship status and general social activity on both trait and state attachment. In Sample I, we assessed time spent alone by asking participants in each EMA whether they were alone at that moment (0 = *with someone*, 1 = *alone*). In Sample II, we determined time spent alone by inquiring whether participants had interacted with others since the previous EMA or during the last one and a half hours when the questionnaire was the first of the day (0 = *yes, in person or virtually*, 1 = *no*). Other potential sociodemographic covariates, including financial strain (Sample I), education level (Sample II), age, gender, and conception via assisted reproduction treatments (Sample II), showed no correlations (*p* > 0.050) with trait attachment anxiety or avoidance in either sample. Thus, these variables were not included as covariates to minimize unnecessary complexity in our statistical models.

In further sensitivity analyses, we also accounted for the average EMA negative and positive emotions. In each EMA of both samples, the participants reported how strongly they experienced four negative (i.e., anger, anxiety, shame, and sadness; omega reliabilities: *ω*_within_ = 0.65–0.67, *ω*_between_ = 0.83–0.90) and four positive (i.e., joy, pride, satisfaction, and excitement; omega reliabilities: *ω*_within_ = 0.83–0.83, *ω*_between_ = 0.89–0.95) emotions at the moment. Participants reported their emotions using a 5‐point Likert scale (1 = *not at all* to 5 = *very much*) in Sample I and a continuous slider scale (0 = *not at all* to 100 = *very much*) in Sample II.

### 2.3 Analytic Strategy

We analyzed our data with DSEM (Asparouhov, Hamaker, and Muthén 2018). The models were estimated in Mplus 8.9–8.10 (Muthén and Muthén 2017). Prior to analysis, we evaluated the stationarity of state attachment variables by conducting Kwiatkowski‐Phillips‐Schmidt‐Shin tests for both mean and trend. These tests and descriptive statistics were computed in R.

DSEM is an analytical combination of time‐series, multilevel, and structural equation modeling (Asparouhov, Hamaker, and Muthén 2018). Compared to the traditional multilevel models used in EMA research, DSEM has several benefits. First, it allows for the simultaneous estimation of numerous fixed and random effects within the same model, unlike the univariate approaches of traditional multilevel models (Asparouhov, Hamaker, and Muthén 2018). Second, DSEM offers robustness against several biases (e.g., negative bias presented in autoregressive effects when using observed mean centering), as it uses latent centering to decompose the total variance of variables into the within‐ and between‐person components (Asparouhov, Hamaker, and Muthén 2018). Third, by providing a flexible framework for estimating random effects and considering the temporal order of observations, DSEM enables the decomposition of each individual's total variance into the components of innovation variance and autoregressive effect (Jongerling, Laurenceau, and Hamaker 2015). This provides separate statistical indicators for the variability and inertia of each state attachment dimension. Finally, DSEM handles missing data and unequal time intervals more sophisticatedly by utilizing the Kalman filter approach (Asparouhov, Hamaker, and Muthén 2018). This provides a better match to the time‐series assumption on equivalent intervals.

**FIGURE 2:**
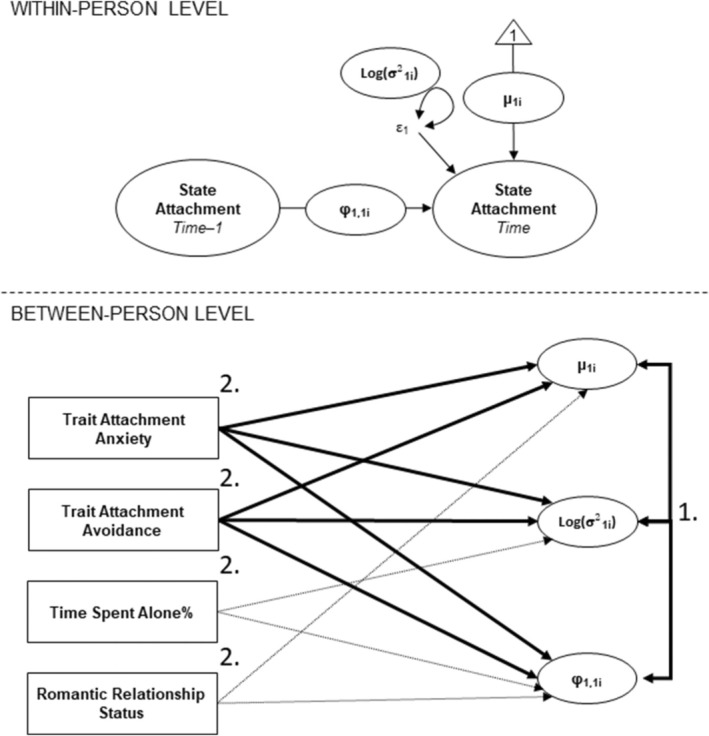
Examining dynamic features of each state attachment dimension with dynamic structural equation model. This strategy was applied separately to state attachment security, avoidance, and anxiety. The sequence of our models is presented with the numbers 1 and 2. At the between‐person level, in the first models (1), we estimated only the covariances between the *baseline* (μ1i), variability (log(*σ* ^2^_1i_)), and inertia (*φ*_1,1i_). In the second models (2), we also estimated the associations of trait attachment anxiety and avoidance (unbroken bolded arrows) as well as time spent alone and romantic relationship status (round dotted arrows) with baseline, variability, and inertia. Variability was estimated using the log transformation to guarantee all individual variances to be positive, which is a standard approach in dynamic structural modeling (Asparouhov, Hamaker, and Muthén 2018). At the within‐person level, *ε*1 and the related circle with bidirectional arrows indicate the fixed effect of innovation variance (with the random effect, log(*σ* ^2^)).

We modeled the baseline, variability, and inertia of each state attachment dimension and the associations of trait attachment with them in separate models for state attachment security, avoidance, and anxiety. Figure 2 illustrates this modeling strategy. In each DSEM, the baseline was modeled by estimating the random intercept/mean, variability by the random innovation variance, and inertia by the random first‐order autoregressive effect. Estimating the random effects allowed each participant to get their own value for baseline, variability, and inertia. At the between‐person level, in the first models, all dynamic features were specified to correlate with each other. This allowed us to address the first research aim by testing the associations between the baseline, variability, and inertia of each state attachment dimension. In the next models, we added trait attachment anxiety, avoidance, time spent alone, and romantic relationship status to predict the baseline, variability, and inertia of each state attachment dimension. This allowed us to address the second research aim by testing the associations of trait attachment with the dynamic features of each state attachment dimension.

In all DSEMs, Bayesian Markov chain Monte Carlo estimation was used with the uninformative priors of Mplus. Two unthinned chains with 50,000 iterations (first 25,000 burn‐in iterations) were used in the estimation. Convergence was checked via the Gelman‐Rubin Proportional Scale Reduction (PSR) and trace plots. If the PSR did not fall below 1.05 in all post‐burn‐in iterations, we doubled the iterations to verify the results. The TINTERVAL command of Mplus was used to specify a 1‐h interval for lag interpretation. An effect was considered as detected if its 95% credible interval (CrI) excluded zero.

To further assess the robustness of our findings, we undertook a series of sensitivity analyses. Regarding our first aim, we conducted non‐preregistered sensitivity analyses to ensure that our results were not merely artifacts influenced by particular distribution patterns in state attachment security and avoidance. Specifically, as in previous research (Bosmans, Van de Walle, et al. 2014; Verhees 2019; Verhees et al. 2023), we observed potential ceiling effects in state attachment security, with participants reporting the highest security level 29.9%–43.3% of the time, and floor effects in state attachment avoidance, with reports peaking at the lowest avoidance level 29.5%–34.1% of the time. Thus, to validate that our findings were not due to these distribution features, we applied a novel two‐part modeling approach specifically designed to address floor and ceiling effects within DSEM (Muthén, Asparouhov, and Shiffman 2024). This approach divided the original state attachment security and avoidance variables into two distinct variables: a binary variable (0/1), where zero signifies the floor (or inverted ceiling) value, and a continuous variable for positive values exceeding the floor.^3^ In this context, scores for state security were inverted, with zero representing the highest level of security (i.e., inverted ceiling value) and six the lowest. We then modeled the correlated random effects for the baseline, variability, and inertia of the continuous variable, in conjunction with the intercept and autoregressive effects of the binary variable, which were permitted to correlate with all random effects. With this two‐part strategy, we were able to thoroughly assess and account for the potential floor and ceiling effects on our results.

Regarding our second aim, we conducted additional non‐preregistered sensitivity analyses in which we controlled for average levels of EMA negative and positive emotions, thus taking into account the connection between daily attachment and emotional processes (Dugan, Khan, and Fraley 2023; Tammilehto et al. 2022, 2023). These DSEMs allowed us to assess whether trait attachment showed incremental predictive power on state attachment dynamics over the general levels of negative and positive emotions.

Finally, as additional preregistered sensitivity analyses for both our research aims, we reconducted all our DSEMs by specifying the time interval according to the intervals of each EMA block in the samples. Thus, state attachment at the previous EMA was treated as the lagged observation of state attachment at the current EMA. Compared to our primary models, these sensitivity analyses aligned with more conventional yet less sophisticated approaches to analyzing EMA data that only used the order of EMA observations instead of exact timestamps. This allowed us to check whether the conclusions would differ when using the more conventional analytical approach to handling EMA observations.

## 3 Results

### 3.1 Preliminary Analyses

Supporting Information 2 outlines the results of the stationarity tests. A majority of tests (ranging from 76.4% to 96.7%) did not yield evidence of non‐stationarity (Table S2). Supporting Information 3 presents the descriptive statistics and correlations for the variables (Table S3). Regarding covariates, being in a romantic relationship was related to higher state attachment security and lower state attachment avoidance, trait attachment anxiety, and trait attachment avoidance across both samples. A greater proportion of time spent alone was associated with lower state attachment security in both samples.

### 3.2 Associations Between Dynamic Features of Each State Attachment Dimension

**FIGURE 3:**
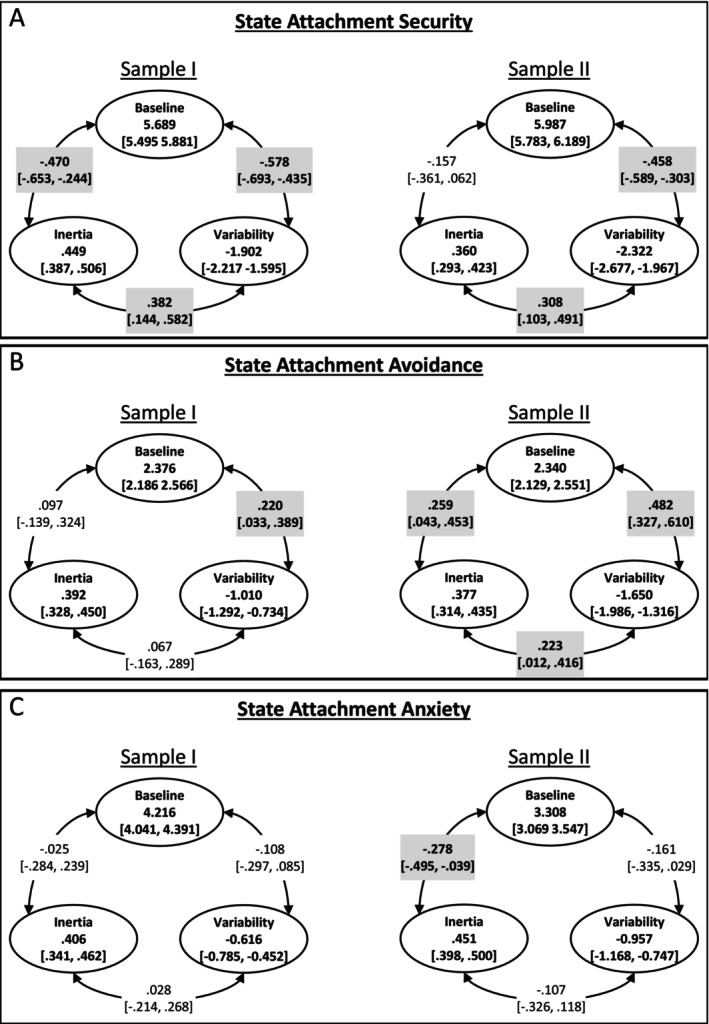
Associations between dynamic features of state attachment security (A), avoidance (B), and anxiety (C). The values in the circles refer to the fixed estimates and their 95% credible intervals (CrIs) for baseline, variability, and inertia. Regarding correlations, the 95% CrIs do not include zero for the values in bold surrounded by a gray area. Variability was estimated using the log transformation to guarantee all individual variances to be positive (Asparouhov, Hamaker, and Muthén 2018).

First, we tested our first hypothesis regarding the associations of high baseline state security with lower variability and higher inertia in state security and then explored the associations for dynamic features of state attachment avoidance and anxiety. Figure 3 displays the relationships between the baseline, variability, and inertia of each state attachment dimension.

For state attachment security (Figure 3A), consistent with our hypothesis, people with high baseline had lower variability in both samples (*r*_Sample I_ = −0.578, *r*_Sample II_ = −0.458, *p*s < 0.001^4^). In Sample I, contrary to our hypothesis, these people with high baseline state security also showed lower (not higher) inertia (*r*_Sample I_ = −0.470, *p* < 0.001). Nevertheless, this result was not replicated in Sample II. Finally, people with high variability exhibited higher inertia in both Sample I (*r*_Sample I_ = 0.382, *p* = 0.002) and Sample II (*r*_Sample II_ = 0.308, *p* = 0.004).

In terms of our exploratory analyses, for state attachment avoidance (Figure 3B), people with high baseline exhibited higher variability in both Sample I (*r*_Sample I_ = 0.220, *p* = 0.022) and Sample II (*r*_Sample II_ = 0.482, *p* < 0.001). In Sample II, they also displayed higher inertia (*r*_Sample I_ = 0.259, *p* = 0.020), but this was not seen in Sample I. Additionally, people with high variability had higher inertia in Sample II (*r*_Sample II_ = 0.223, *p* = 0.040), but this was not evident in Sample I. Finally, for state attachment anxiety (Figure 3C), we observed only one association. In Sample II, people with high baseline had lower inertia (*r*_Sample II_ = −0.278, *p* = 0.024), but this was not replicated in Sample I.

Supporting Information 4 presents the sensitivity analyses for the first research aim on the associations of dynamic state attachment features. Figure S4A depicts the results of the non‐preregistered analyses using a two‐part modeling approach to address potential floor and ceiling effects in state attachment. Since state anxiety did not exhibit any indications of floor or ceiling effects (Supporting Information 3 and Figure 3), these sensitivity analyses were conducted exclusively for state security (inverted) and avoidance. Across both samples, these analyses reaffirmed the observed associations between baseline and variability in state attachment security and avoidance. Further, in Sample I, the association of high baseline with low inertia in state security was confirmed. However, the link between high inertia and variability in state security was not found in either Sample I or II. Further, for state avoidance, the associations of high inertia with high baseline and variability were not replicated in Sample II. Finally, a negative link emerged between inertia and variability of state avoidance in Sample I. Overall, these results indicated that floor and ceiling effects could not account for the most robust patterns observed in our primary analyses. Thus, our main findings regarding the associations between baseline and variability in state security and avoidance were robust against floor and ceiling effects. Specifically, these findings supported our first hypothesis about the link between high baseline and low variability in state security.

Figure S4B depicts the preregistered sensitivity analyses where state attachment at the previous EMA was used as the lagged observation. These analyses yield findings similar to those of primary analyses. The notable exception was that in state avoidance, the associations of inertia with baseline and variability were not observed in Sample II. Nevertheless, again, our main findings regarding the associations between baseline and variability in state security and avoidance proved robust across modeling varying time intervals.

### 3.3 Associations of Trait Attachment With Dynamic Features of State Attachment

Table 1 presents the standardized effects of trait attachment anxiety and avoidance on the dynamic features of state attachment security, avoidance, and anxiety. Supporting Information 5 presents the unstandardized effects (Table S5).

**TABLE 1:** Standardized associations of trait attachment with dynamic features of state attachment.

Predictors	Baseline of attachment state		Variability of attachment state		Inertia of attachment state	
	Sample I	Sample II	Sample I	Sample II	Sample I	Sample II
	Posterior Mdn β* [95% CrI]	Posterior Mdn β* [95% CrI]	Posterior *Mdn* β* [95% CrI]	Posterior Mdn β* [95% CrI]	Posterior Mdn β* [95% CrI]	Posterior Mdn β* [95% CrI]
*Model for state attachment security*						
Trait attachment anxiety	**−0.185** **[−0.320, −0.045]**	**−0.262** **[−0.380, −0.134]**	**0.271** **[0.132, 0.397]**	**0.186** **[0.055, 0.306]**	0.050 [−0.123, 0.221]	0.040 [−0.120, 0.197]
Trait attachment avoidance	**−0.225** **[−0.355, −0.088]**	−0.137 [−0.280, 0.010]	0.022 [−0.110, 0.158]	**0.222** **[0.073, 0.354]**	0.171 [−0.002, 0.334]	0.012 [−0.173, 0.193]
% Time spent alone	0.009 [−0.134, 0.147]	**−0.220** **[−0.334, −0.097]**	−0.097 [−0.235, 0.046]	0.067 [−0.056, 0.188]	0.032 [−0.152, 0.212]	−0.038 [−0.186, 0.113]
Romantic relationship status	**0.187** **[0.035, 0.327]**	0.084 [−0.065, 0.233]	−0.100 [−0.243, 0.050]	0.136 [−0.019, 0.273]	0.037 [−0.155, 0.221]	−0.007 [−0.191, 0.177]
*R* ^2^	0.137	0.159	0.109	0.118	0.061	0.027
*Model for state attachment avoidance*						
Trait attachment anxiety	0.114 [−0.028, 0.254]	0.077 [−0.054, 0.206]	0.113 [−0.029, 0.250]	0.061 [−0.071, 0.190]	−0.144 [−0.315, 0.035]	0.010 [−0.149, 0.168]
Trait attachment avoidance	**0.264** **[0.126, 0.388]**	**0.342** **[0.192, 0.469]**	0.028 [−0.110, 0.167]	**0.207** **[0.052, 0.345]**	0.040 [−0.138, 0.209]	0.121 [−0.071, 0.301]
% Time spent alone	0.031 [−0.115, 0.175]	−0.028 [−0.147, 0.096]	**−0.162** **[−0.301, −0.014]**	−0.018 [−0.141, 0.109]	0.119 [−0.070, 0.298]	−0.031 [−0.180, 0.118]
Romantic relationship status	−0.030 [−0.181, 0.120]	0.011 [−0.143, 0.158]	−0.017 [−0.168, 0.135]	0.113 [−0.044, 0.258]	0.016 [−0.178, 0.205]	0.016 [−0.177, 0.206]
*R* ^2^	0.102	0.137	0.056	0.072	0.062	0.038
*Model for state attachment anxiety*						
Trait attachment anxiety	**0.294** **[0.162, 0.407]**	**0.282** **[0.154, 0.396]**	0.092 [−0.055, 0.235]	0.125 [−0.009, 0.251]	−0.079 [−0.269, 0.113]	−0.027 [−0.213, 0.151]
Trait attachment avoidance	**−0.225** **[−0.340, −0.097]**	**−0.212** **[−0.346, −0.065]**	0.029 [−0.115, 0.172]	0.103 [−0.054, 0.247]	0.007 [−0.176, 0.187]	0.098 [−0.107, 0.290]
% Time spent alone	**−0.220** **[−0.343, −0.083]**	**−0.137** **[−0.254, −0.016]**	−0.082 [−0.233, 0.069]	−0.023 [−0.148, 0.104]	−0.012 [−0.203, 0.186]	0.061 [−0.097, 0.215]
Romantic relationship status	−0.113 [−0.247, 0.031]	−0.055 [−0.200, 0.096]	0.060 [−0.100, 0.214]	**0.229** **[0.073, 0.368]**	−0.156 [−0.341, 0.044]	0.140 [−0.066, 0.328]
*R* ^2^	0.211	0.160	0.038	0.091	0.059	0.058

*Note:* In Sample I, *N*_participants_ = 122, *N*_observations_ = 4629. In Sample II, *N*_participants_ = 127, *N*_observations_ = 5322. For the values in bold, the 95% credible interval (95% CrI) does not contain zero. The results were summarized in R using the MplusAutomation package (Hallquist and Wiley 2018).

First, we tested our second hypothesis regarding the links of low trait attachment anxiety and avoidance with the higher baseline, lower variability, and higher inertia of state security. As hypothesized, low trait attachment anxiety predicted higher baseline state security in Sample I (β*_Sample I_ = −0.185, *p* = 0.010) and Sample II (β*_Sample II_ = −0.262, *p* < 0.001). In line with our hypothesis, low trait attachment anxiety also predicted lower variability of state security in Sample I (β*_Sample I_ = 0.271, *p* < 0.001) and Sample II (β*_Sample II_ = 0.186, *p* = 0.006). Moreover, partially in line with our hypothesis, low trait attachment avoidance predicted higher baseline state security in Sample I (β*_Sample I_ = −0.225, *p* = 0.002). While this association was in the same direction in Sample II, the 95% CrI excluded the zero (β*_Sample II_ = −0.137, *p* = 0.070). Finally, low trait attachment avoidance predicted lower variability of state security in Sample II (β*_Sample II_ = 0.222, *p* = 0.004), but not in Sample I, thus providing only partial support for our hypothesis. Contrary to our hypotheses, no links of trait attachment were detected with the inertia of state security in either sample.

Second, we tested our third and fourth hypotheses linking trait attachment dimensions to their corresponding baselines of state avoidance and anxiety and explored their associations with other dynamic features of the insecure states. Consistent with our hypothesis, high trait avoidance predicted higher baseline state avoidance in both samples (β*_Sample II_ = 0.264, β*_Sample II_ = 0.342, *p*s < 0.001). It also predicted higher variability of state avoidance in Sample II (β*_Sample II_ = 0.207, *p* = 0.008), though this effect was not replicated in Sample I. No links were found between trait attachment and the inertia of state avoidance.

Similarly, in line with our hypothesis, high trait anxiety predicted higher baseline state anxiety in both samples (β*_Sample II_ = 0.294, β*_Sample II_ = 0.282, *p*s < 0.001). Additionally, as a robust exploratory finding, high trait avoidance predicted lower baseline state anxiety in Sample I (β*_Sample I_ = −0.225, *p* < 0.001) and Sample II (β*_Sample II_ = −0.212, *p* = 0.006). No links were found between trait attachment and the variability or inertia of state anxiety.

Supporting Information 6 presents the sensitivity analyses on the associations of trait attachment with state attachment dynamic features. Tables S6A and S6B show the results of non‐preregistered analyses examining the associations of trait attachment after accounting for the average EMA negative and positive emotions. Even with the emotions considered, all detected associations of both trait dimensions remained robust in Sample I. In Sample II, the observed associations persisted with the baselines of state attachment anxiety and avoidance. However, the association of trait attachment avoidance was not detected with state avoidance variability. Moreover, the links of the trait attachment dimensions with the baseline and variability of state security became non‐significant. Further analyses on Sample II revealed that both high negative (*β* = −0.114, 95% CrI, [−0.201, −0.049]) and low positive emotions (*β* = −0.077, 95% CrI, [−0.151, −0.025]) mediated the associations of high trait attachment anxiety with lower baseline state security. Low positive emotions also mediated the association of high trait attachment anxiety with higher variability of state security (*β* = 0.113, 95% CrI, [0.024, 0.251]). Similarly, low positive emotions mediated the associations of high trait avoidance with lower baseline (*β* = −0.108, 95% CrI, [−0.209, −0.037]) and higher variability (*β* = 0.160, 95% CrI, [0.034, 0.350]) of state security. Thus, while our findings in Sample I remained robust after covarying emotions, in Sample II, variations in emotions accounted for the links of trait attachment dimensions with the baseline and variability of state security.

Finally, Tables S6C and S6D provide the results of sensitive analyses, in which we treated state attachment at the previous EMA as the lagged observation. We observed only minor absolute differences compared to the main results, and the interpretations regarding the robustly detected associations remained consistent. Yet, as notable deviations from the interpretations of the main analyses, trait avoidance predicted higher inertia of state security in Sample I, while trait attachment anxiety predicted higher variability of state anxiety in Sample II. Yet, since these links were not observed in our main analyses, it is prudent to refrain from drawing conclusions until they are robustly replicated in future research.

### 3.4 Simulations on Statistical Power

Lastly, we conducted Monte Carlo simulations to assess the smallest effect sizes our study design could detect. In the simulations, we used the same DSEM, sample, and missing data structure as in our primary analyses. The population correlations between trait attachment and covariates were specified using the correlation structure in our data. Similarly, the population models for all random effects were based on the estimates of our conducted DSEMs. The simulations with 500 replications indicated that the smallest detectable correlations exceeding 0.80 power between the dynamic features of each state attachment dimension were *r* = |0.300|–|0.360| in Sample I and *r* = |0.280|–|0.360| in Sample II. The standardized estimates exceeding 0.80 power for the associations of trait attachment dimensions in Sample I and II were |0.310|–|0.320| and |0.300|–|0.350| with baselines, |0.300|–|0.310| and |0.290|–|0.330| with variabilities, and |0.360|–|0.380| and |0.360|–|0.400| with inertias, respectively. Thus, our designs were able to detect medium‐sized effects, while the power to detect small effects was more limited.

## 4 Discussion

The attachment system, a dynamic and context‐sensitive motivational system, has been underexplored in real‐time everyday settings. Our EMA study addressed this gap by investigating (a) the associations between dynamic state attachment features and (b) the links between trait attachment and these features with the aim of enhancing understanding of the attachment system functioning in daily life. Our findings revealed several robust patterns across two adult samples and sensitivity analyses. As hypothesized, individuals with high baseline state security exhibited lower variability in their state security. Moreover, our exploratory analyses revealed that, for state avoidance, those with high baseline showed greater variability. However, we found no support for our hypothesis linking high baseline state security to higher inertia in state security. When examining trait attachment, our results supported most of our hypotheses. First, low trait attachment anxiety and avoidance predicted higher baseline and lower variability of state security. Second, high trait avoidance predicted higher baseline state avoidance, and high trait anxiety predicted higher baseline state anxiety. Our exploratory analyses further revealed that high trait avoidance predicted a lower state anxiety baseline in both samples. However, we found no support for our hypothesis linking trait attachment to the inertia of state security.

### 4.1 Associations Between Dynamic Features Within Each State Attachment Dimension

According to attachment theory, a well‐established sense of security directs individuals' interpretations and reactions in various situations (Bosmans et al. 2020; Mikulincer and Shaver 2023). Consistent with this and a previous diary study on children (Bosmans, Van de Walle, et al. 2014), we found support for our hypothesis that people with high baseline state security are less susceptible to deviations in their sense of security, even after accounting for potential ceiling effects. This reduced variability may be partly attributed to their tendency to assimilate information in ways that affirm their secure beliefs and expectations (Bosmans et al. 2020; Bosmans, Van de Walle, et al.  2014). Such secure‐congruent information processing biases sustain trust in the attachment figures' actual and symbolic availability (Dykas and Cassidy 2011). Thus, the link between high baseline state security and low variability may indicate the fundamental steadiness of the attachment system functioning in those with a well‐established sense of security. Their attachment system may be less prone to activation by daily attachment‐related stimuli, particularly those with ambiguous implications. Consequently, a strong sense of security can lead to self‐fulfilling patterns of interpreting and engaging with daily situations.

In addition to information processing biases, several other evocative and active interaction processes with the environment could explain the association between high baseline state security and its low variability. Specifically, individuals with a strong sense of security may naturally exhibit behaviors that evoke fewer threatening reactions from those around them (Donnellan et al. 2008). Furthermore, they may often take proactive steps toward establishing and maintaining relationships and environments that are both safe and stable (Donnellan et al. 2008). These evocative and active processes may create a self‐sustaining cycle, where secure individuals strive toward nurturing environments that enhance their sense of security (Mikulincer and Shaver 2023; Tammilehto et al. 2022). Such dynamics may become particularly salient in adulthood due to the growing significance of individual agency in shaping perceptions of oneself and others (Fraley and Roisman 2019).

Against our hypothesis, high baseline state security was also associated with lower, not higher, inertia of state security in Sample I, even after accounting for a potential ceiling effect. Though not replicated across samples, this preliminary finding is interesting as it deviates from a child diary study reporting an association between high baseline and high inertia of state security (Verhees 2019). This discrepancy between children and adults may reflect divergent developmental manifestations of everyday attachment dynamics. In childhood, a strong sense of security may be related to more stable and context‐insensitive appraisals regarding the attachment figure's availability. In turn, adults with high baseline state security may exhibit more context‐sensitive appraisals of attachment figure availability due to their more multifaceted and complex attachment representations (Fraley 2007). As a result, their attachment systems might show elevated functional flexibility, effectively restoring the sense of security to the baseline following perturbations.

Compared to security, our exploratory analyses on state avoidance revealed an opposite relationship between baseline and variability. Specifically, individuals with high baseline state avoidance exhibited greater variability in state avoidance. This finding remained robust even after accounting for potential floor effects, generating novel hypotheses for future research. It suggests that people with high baseline state avoidance may rapidly oscillate between attempts to deactivate painful attachment experiences and their reactivation. Furthermore, although not robust for floor effects, both high baseline and high variability were linked to higher inertia in Sample II. This overall pattern suggests that people with high baseline state avoidance may experience more pronounced fluctuations in state avoidance, after which it may also take longer for them to reestablish their baseline. This interpretation aligns with research suggesting that, under stress, deactivating strategies may lead to prolonged distress, making recovery challenging (Mikulincer, Dolev, and Shaver 2004). Further research is required to confirm and expand upon these initial interpretations of our exploratory findings.

Finally, regarding state anxiety, the only exploratory finding was a link between high baseline and low inertia (only in Sample II). The lack of more robust patterns may suggest that people with high baseline state anxiety show highly unpredictable state attachment responses. This could arise from their expectations that intimate others are highly inconsistent in their availability, coupled with a related hyperactivation coping style (Arriaga et al. 2018; Bosmans et al. 2020). Alternatively, however, the lack of associations may be due to the low reliability of our state anxiety scales, particularly at the within‐person level (i.e., 0.41–0.47). This raises concerns about whether the content of our state anxiety items sufficiently captured the nuances of the dynamic anxious attachment experiences. Albeit all reliabilities conformed to the benchmarks in the EMA literature (Nezlek 2017), future research could handle the issue by using a higher number of items to assess state anxiety. However, this approach is known to increase the burden on the participants (Eisele et al. 2022). Thus, researchers must meticulously design studies to balance the need for enhanced reliability through multiple items against the need to minimize the reporting burden.

### 4.2 Role of Trait Attachment in Dynamic Features of State Attachment

As hypothesized, people with low trait attachment anxiety (both samples) and low avoidance (one of the samples) exhibited higher baseline and lower variability of state security. Further supporting our hypotheses, high levels of trait attachment anxiety and avoidance were consistently associated with their respective high baselines of state attachment anxiety and avoidance. These findings resonate with our other findings, which linked high baseline state security to its lower variability. On one hand, trait attachment can direct people's interpersonal perceptions and appraisals of attachment figures' availability and threats (Dykas and Cassidy 2011). On the other hand, it can guide specific behaviors that build one's socioemotional contexts and evoke responses in others (Donnellan et al. 2008). Specifically, prototypical anxious and avoidance representations likely skew information processing and interpersonal behaviors, leading to either chronic hyperactivation or deactivation of the attachment system (Mikulincer and Shaver 2023). Furthermore, prototypical insecure representations seem to predispose people to interpretations and behaviors that induce volatile and erratic shifts in their sense of security, whereas the opposite applies to prototypical secure representations. The functional insecure patterns may partly but not entirely arise from escalated negative and blunted positive emotions, as indicated by our sensitivity analyses and corroborated by other research (Dugan, Khan, and Fraley 2023). Thus, our trait attachment findings not only bolster the interpretation that a high steadiness in state security is integral for a well‐established sense of security but also emphasize that the key characteristic of prototypical insecure representations is a marked instability in the sense of security.

In contrast, trait attachment anxiety and avoidance were not associated with the inertia of state security. While these null findings contrast with our hypotheses, they are consistent with our previous study, in which we found no associations between trait attachment and inertia of positive and negative emotions in the same samples (Tammilehto et al. 2023). Thus, it may be that the resistance to change in mental states is not the central dynamic feature through which trait attachment manifests in daily life. However, the null findings may also relate to the demands of statistical power to detect associations with inertia. Our simulations suggested that our design was more powerful in studying baseline and variability than inertia. Thus, future studies with larger samples are required to inspect further whether trait attachment meaningfully manifests in the inertia of mental states.

Finally, as a robust exploratory finding, we discovered that high trait avoidance was associated with lower baseline state anxiety in both samples. This finding deviates from the positive association commonly observed between attachment avoidance and anxiety at the trait level (Del Giudice 2011), highlighting the unique insights that research on state attachment dynamics can offer. It may suggest that individuals with high trait avoidance tend to suppress the (hyper)activation of their attachment system in daily life. It is further interesting to note that the relationship between avoidance and anxiety was not symmetrical, as trait anxiety was not associated with the baseline state avoidance. This asymmetry underscores the power of motivation to avoid psychological pain associated with needing love and care while anticipating rejection (Bowlby 1980; Mikulincer and Shaver 2023). Thus, all else being equal, the trait‐like motivation for deactivation might surpass the trait‐like motivation for hyperactivation in everyday contexts. This tentative hypothesis requires further testing in future research.

### 4.3 General Discussion

Overall, our study provides new insights into the real‐time regulatory dynamics of the attachment system. This marks a significant step forward in our understanding of how the attachment system functions. First, people with high baseline state security experience fewer and less intense ups and downs in their sense of security. This steadiness likely reflects their equanimity in responding to ambiguous attachment cues and potential false alarms, which enables them to maintain their daily routines without disruption and conserve mental resources for non‐attachment activities (Mikulincer and Shaver 2023). Second, people with high baseline state avoidance are more susceptible to experiencing escalated deviations in their sense of avoidance. This instability may reflect the inherent fragility of deactivation strategies to collapse under daily stress (Mikulincer, Dolev, and Shaver 2004). These findings contribute to attachment models that acknowledge the dynamism of attachment representations and their impact on socioemotional processes (Arriaga et al. 2018; Bosmans et al. 2020). Looking ahead, exploring the interaction processes between attachment states and environmental responses can help understand how individuals maintain and reinforce their daily (in)security.

Moreover, our findings suggest that trait attachment anxiety and avoidance are significantly linked to the baselines of each state attachment dimension, as well as to the variability in state security. Yet, trait attachment alone does not explain a significant portion of the variance, suggesting that other factors also play crucial roles in the dynamic features of state attachment. At the intrapersonal level, these may include self‐regulatory abilities related to emotion regulation (Tammilehto et al. 2022) and cognitive control (Lindblom and Bosmans 2022). At the interpersonal level, one's attachment figures' own trait attachment and related abilities to express warmth, love, and validation may play a role (Arriaga et al. 2018; Kaurin, Pilkonis, and Wright 2022). Crucially, the amount of unexplained variance underscores the promise that research on state attachment holds for elucidating the nuances of individual differences beyond what is captured by solely assessing trait attachment. Thus, exploring the predictive value of the dynamic features on socioemotional outcomes offers a promising avenue for uncovering insights into the role of attachment processes in behaviors and well‐being.

Finally, our findings introduce new ideas for attachment‐based therapies that aim to shift prototypical insecure representations to more secure ones (e.g., Slade and Holmes 2019). According to the learning theory of attachment, stable changes in trait attachment are preceded by periods of increased variability in state attachment, indicating that the attachment system is approaching a tipping point for changes in its functioning (Bosmans et al. 2020). Our findings suggest that people with high trait attachment anxiety and avoidance experience heightened variability in state security, indicating that their attachment system may be relatively destabilized by default. This destabilization suggests that transitions from trait‐like insecurity to security are potentially more common than shifts from security to insecurity, given that heightened variability may be an early marker of the system reorganization (Bosmans et al. 2020). Such a functional pattern would align with the attachment system's set goal of attaining and maintaining a sense of security whenever possible (Mikulincer and Shaver 2023). Future studies employing multi‐wave EMA designs could test this hypothesis and potential mechanisms destabilizing the attachment system.

### 4.4 Limitations

Our study has several limitations. First, despite using two samples with diverse demographic distributions, our power simulations suggested they were limited to detecting small effects. Moreover, the predominance of females in our samples limits the generalizability of our findings to males. While women exhibit higher trait anxiety and lower avoidance (Del Giudice 2011), the extent to which gender shapes state attachment dynamics—both independently and in conjunction with trait attachment—remains unclear. Additionally, compared to our Western samples, adult attachment processes may differ in their daily manifestations in non‐Western populations due to varying social norms and structures, which may significantly influence how individuals maintain attachment beliefs and expectations (Thompson, Simpson, and Berlin 2022). Second, the exact reasons for some non‐replicated findings between the samples are difficult to recognize. These disparities might be attributed to variations in age and gender, or they could simply be due to random sampling error. Finally, as already discussed, our state anxiety scales showed low within‐person reliability. Relatedly, a significant limitation in our statistical approach was not modeling the measurement error, which may have especially influenced the estimates of variability and inertia. In future studies with a larger number of EMA observations, researchers may use approaches developed to model measurement errors with DSEM (Schuurman and Hamaker 2019).

### 4.5 Conclusions

Attachment theory has long emphasized the dynamic responses of the attachment system to threats and the availability of attachment figures (Ainsworth et al. 1978; Bowlby 1982). Our EMA study represents a comprehensive investigation into the dynamic features of state attachment and the associations of relatively stable trait attachment with these features. Our findings underscore the significance of baseline and variability in comprehending attachment system dynamics in everyday life. We hope these findings will inspire future research to explore the impact of these dynamic features on individuals' socioemotional well‐being, vulnerability, resilience, and thriving. By further examining these aspects, researchers can solidify the role of the attachment system as a key driver of people's crucial goals and motives in their everyday lives.

## 5 Author Contributions

**Jaakko Tammilehto**: Conceptualization (lead), Data Curation (lead), Formal Analysis (lead), Funding Acquisition (lead; Finnish Cultural Foundation), Investigation (lead), Methodology (lead), Project Administration (lead), Validation (lead), Visualization (lead), Writing – Original Draft Preparation (lead), Writing – Review & Editing (lead). **Aleksandra Kaurin**: Conceptualization, Investigation, Writing – Review & Editing. **Guy Bosmans**: Conceptualization, Investigation, Writing – Review & Editing. **Peter Kuppens**: Conceptualization, Investigation, Writing – Review & Editing. **Marjo Flykt**: Conceptualization, Investigation, Funding Acquisition, Writing – Review & Editing. **Mervi Vänskä**: Conceptualization, Investigation, Funding Acquisition, Writing – Review & Editing. **Kirsi Peltonen**: Conceptualization, Investigation, Writing – Review & Editing. **Jallu Lindblom**: Conceptualization, Data Curation, Funding Acquisition (lead; Jenny & Antti Wihuri Foundation), Investigation, Methodology, Project Administration, Supervision (lead), Writing – Review & Editing.

## Conflicts of Interest

The authors declare no conflicts of interest.

## Supporting information


Data S1.

## Data Availability

The data of Sample I and its codebook can be found at https://osf.io/3jhvt. The data of Sample II is not shared to protect the privacy of the participants in the ongoing longitudinal study.

## Endnotes

^1^
For preregistration, see https://osf.io/kvyxh. All analysis scripts of both samples can be found at https://osf.io/qke5m. The data of Sample I and its codebook can be found at https://osf.io/3jhvt. The data of Sample II is not shared to protect the privacy of the participants in the ongoing longitudinal study.

^2^
Regarding Sample I, the results have already been reported and interpreted in detail in another study using the same sample (Tammilehto et al. 2022). The data of Sample I used in the multilevel confirmatory factor analyses can be found at https://osf.io/e8423. The data of Sample II is not shared to protect the privacy of the participants in the ongoing longitudinal study.

^3^
When the binary variable indicated a floor value (zero), the continuous variable was treated as missing.

^4^
All reported *p*‐values for point estimates are two‐tailed Bayesian *p*‐values (i.e., 2 × one‐tailed *p*‐value).
