# Supplementary material for: Everyday State Attachment: Dynamic Features and Role of Trait Attachment
Source: J Pers. 2024 Oct 17;93(3):781–95. doi: 10.1111/jopy.12975 (PMC12053816; doi:10.1111/jopy.12975)
Supplement: Supplementary file 1 — Data S1. [file JOPY-93-781-s001.docx]

**Supporting Information for Everyday State Attachment: Dynamic Features and Role of Trait Attachment**

Jaakko Tammilehto^1,2^, Aleksandra Kaurin^3^, Guy Bosmans^4^, Peter Kuppens^4^, Marjo Flykt^1, 2^, Mervi Vänskä^1^, Kirsi Peltonen^5^, and Jallu Lindblom^1^

^1^ Faculty of Social Sciences / Psychology, Tampere University

^2^ Department of Psychology and Logopedics, Faculty of Medicine, University of Helsinki

^3^ Clinical Child and Adolescent Psychology and Psychotherapy, University of Wuppertal

^4^ Faculty of Psychology and Educational Sciences, KU Leuven

^5^ INVEST Research Flagship Center, University of Turku

**Table of Contents**

[**Supporting Information 1: Multilevel Confirmatory Factor Analysis for the Measurement Model of State Adult Attachment Measure 2**](#_Toc177987290)

[**Supporting Information 2: Testing Stationarity of State Attachment 6**](#_Toc177987291)

[**Supporting Information 3: Descriptive Statistics and Correlations of Study Variables 8**](#_Toc177987292)

[**Supporting Information 4: Sensitivity Analyses for Associations of Dynamic Features Within Each State Attachment Dimension 10**](#_Toc177987293)

[**Supporting Information 5: Unstandardized Associations of Trait Attachment with Dynamic Features of State Attachment 13**](#_Toc177987294)

[**Supporting Information 6: Sensitivity Analyses for Associations of Trait Attachment with Dynamic Features of State Attachment 15**](#_Toc177987295)

[**References 20**](#_Toc177987296)

## Supporting Information 1: Multilevel Confirmatory Factor Analysis for the Measurement Model of State Adult Attachment Measure

**Figure S1**

*Tested Measurement Model for State Attachment Dimensions*


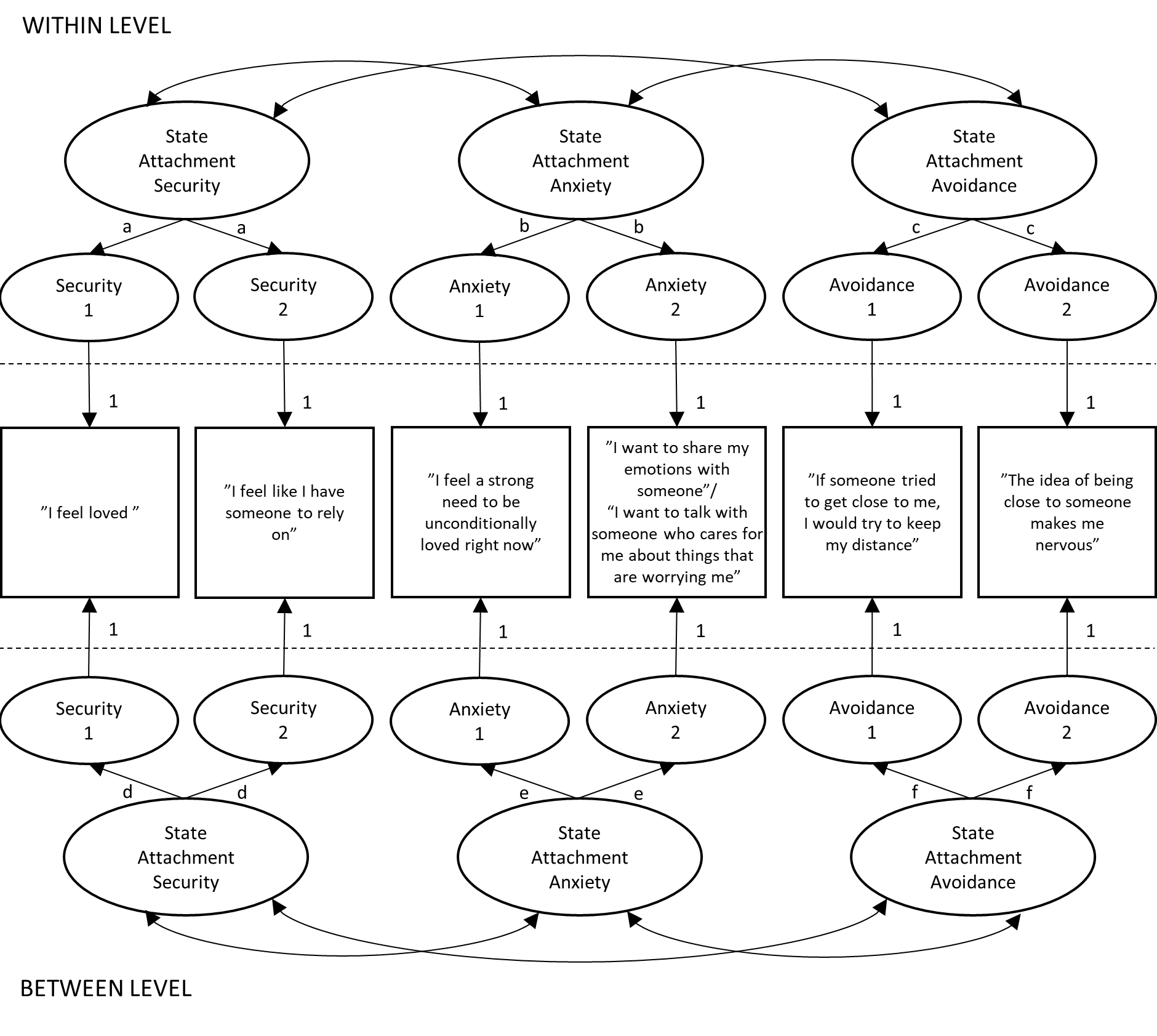


*Notes.* This figure is based on published Figure 1 in Tammilehto et al. (2022) and is adapted here under the Creative Commons Attribution License (CC BY 4.0), accessible at http://creativecommons.org/licenses/by/4.0/. The indicator “I want to share my feelings with someone” was used in Sample I, whereas the indicator “I want to talk with someone who cares for me about things that are worrying me” was used in Sample II. In Sample I, all unique variances were estimated freely. However, in Sample II, we fixed the unique between-person variances of security indicators to be equal. This was because the security indicator ”I feel like I have someone to rely on” showed negative residual variance at the between-person level when we estimated this variance without constraints.

| **Table S1A**  *Testing the Three-Factor Model of State Attachment Security, Avoidance, and Anxiety* | | | | | | | |  |
| --- | --- | --- | --- | --- | --- | --- | --- | --- |
| Model | *df* | Scaled  χ^2^ | CFI | RMSEA | SRMR  within/ between | *AIC* | *BIC* | |
| *Sample I* |  |  |  |  |  |  |  | |
| Within-person level model | 9 | 126.79 | .953 | .077 | .056/.008 | 74859.75 | 75110.91 | |
| Between-person level model | 9 | 27.93 | .995 | .024 | .000/.109 | 74631.25 | 74882.41 | |
| Within- and between-person level model | 18 | 177.77 | .949 | .057 | 056/.109 | 74878.53 | 75071.73 | |
| *Sample II* |  |  |  |  |  |  |  | |
| Within-person level model | 9 | 18.53 | .995 | .020 | .016/.002 | 83379.47 | 83636.07 | |
| Between-person level model | 10 | 30.63 | .995 | .021 | .000/.067 | 83371.98 | 83622.01 | |
| Within- and between-person level model | 19 | 46.20 | .990 | .020 | .016/.069 | 83391.93 | 83582.93 | |
| *Notes.* In Sample I, *N_participants_* = 122, *N_observations_ =* 4629*.* In Sample II, *N_participants_* = 127, *N_observations_ =* 5322*.* The full information maximum likelihood estimation with robust standard errors and the Yuan-Bentler scaled test statistic was used as an estimator. Figure S1 presents the theoretical structure of the SAAM measurement model. The specified structure was the same for both within-person and between-person levels. The latent variables of state attachment security, anxiety, and avoidance had two indicators each, and the loadings of both indicators within each latent variable were fixed to the same value. The aim of fixing the loadings to the same value was to increase the correspondence to the average scores used in the main analyses and guarantee empirical model identification. Moreover, the variances of the latent variables were fixed to one, and the latent variables were specified to correlate. Finally, all residual covariances of the indicators were fixed to zero. Notably, in Sample II, we yet fixed the unique variances of state attachment security items to the same value at the between-person level. This was because the security indicator ”I feel like I have someone to rely on” showed negative unique variance at the between-person level when we estimated this variance without constraints.  In testing the SAAM measurement model, we used a three-stage strategy suggested by Sadikaj et al. (2021). First, we tested a model in which the structure at the within-person level was specified in line with the three-factor structure of the SAAM, whereas the structure at the between-person level was saturated (i.e., all indicators covary with each other). This allowed us to assess fit at the within-person level. Second, we tested a model in which the structure at the between-person level was specified in line with the three-factor structure, whereas the structure at the within-person level was saturated. This allowed us to assess fit at the between-person level. Finally, to assess overall model fit, we tested a model in which the structure at both within-person and between-person levels was specified in line with the three-factor structure. The robust comparative fit index (CFI; Brosseau-Liard & Savalei, 2014), robust root mean square error of approximation (RMSEA; Brosseau-Liard et al., 2012), and standardized root mean square residual (SRMR) were used to assess model fit. The benchmarks of adequate fit were CFI > .95, RMSEA < .06, and SRMR < .08 (Hu & Bentler, 1999). Notably, the results regarding Sample I have been previously published and discussed in detail in Supplemental Material 1 of Tammilehto et al. (2022). CFI = robust comparative fit index; RMSEA = robust root-mean-square error of approximation; SRMR = standardized root mean square residual; * *p* < 0.001 | | | | | | | | |

| **Table S1B**  *Testing Cross-Level Metric Invariance of Attachment States* | | | | | | | | | | |
| --- | --- | --- | --- | --- | --- | --- | --- | --- | --- | --- |
| Sample I | *df* | Scaled  χ^2^ | CFI | RMSEA | SRMR  within/ between | Scaled  ∆χ^2^ test | *p* | | *AIC* | *BIC* |
| Original model with freely estimated factor loadings (Figure S1) | 18 | 177.77 | .949 | .057 | .056 /.109 |  |  | | 74878.53 | 75071.73 |
| Model with cross-level metric invariance in all attachment states | 21 | 227.56 | .935 | .059 | .056/.325 | 52.57 | < .001 | | 74949.36 | 75123.24 |
| Model with cross-level metric invariance in state attachment anxiety and avoidance | 20 | 181.30 | .946 | .056 | .056 /.160 | 7.83 | .020 | | 74893.37 | 75073.69 |
| Model with cross-level metric invariance in state attachment anxiety | 19 | 176.55 | .948 | .055 | .056/.106 | 1.53 | .216 | | 74880.05 | 75066.82 |
| Sample II | *df* | Scaled  χ^2^ | CFI | RMSEA | SRMR  within/ between | Scaled  ∆χ^2^ test | | *p* | *AIC* | *BIC* |
| Original model with freely estimated factor loadings (Figure S1) | 19 | 46.20 | .990 | .020 | .016/.069 |  | |  | 83391.93 | 83582.74 |
| Model with cross-level metric invariance in all attachment states | 22 | 345.00 | .915 | .056 | .027/.512 | negative | |  | 83701.18 | 83872.25 |
| Model with cross-level metric invariance in state attachment anxiety and avoidance | 21 | 133.41 | .958 | .040 | .021/.245 | 63.77 | | < .001 | 83529.21 | 83706.86 |
| Model with cross-level metric invariance in state attachment anxiety | 20 | 85.55 | .977 | .030 | .016/.138 | 90.36 | | < .001 | 83445.82 | 83541.07 |
| Model with cross-level metric invariance in state attachment avoidance | 20 | 94.31 | .971 | .033 | .020/.212 | 20.23 | | < .001 | 83475.84 | 8366. 07 |
| *Notes.* In Sample I, *N_participants_* = 122, *N_observations_ =* 4629*.* In Sample II, *N_participants_* = 127, *N_observations_ =* 5322*.* The cross-level metric invariance constituted a test of whether state attachment dimensions at the between-person level can be interpreted as an aggregate of its within-person level counterpart (Jak, 2019; Jak & Jorgensen, 2017). Thus, testing the cross-level metric invariance allowed us to assess whether the interpretations of the state attachment dimensions are similar at the within- and between-person levels (Jak & Jorgensen, 2017). The cross-level metric invariance of state attachment dimensions was assessed by comparing the original model (Figure S1) to the models in which all or some loadings were fixed to be equal across within- and between-person levels. In the comparisons of the models, we used the scaled chi-square difference test with α = 0.050 (Satorra & Bentler, 2001), Akaike's Information Criteria (AIC), and Bayesian Information Criteria (BIC). In AIC and BIC, smaller values indicate better model fit. We used a difference of ≥ \|2.00\| to indicate the meaningful difference in AIC and BIC values (Burnham & Anderson, 2004). In the scaled ∆χ^2^ tests, models were compared to the original model with freely estimated factor loadings. Notably, the results regarding Sample I have been previously published and discussed in detail in Supplemental Material 1 of Tammilehto et al. (2022). CFI = robust comparative fit index; RMSEA = robust root-mean-square error of approximation; SRMR = standardized root mean square residual. | | | | | | | | | | |

## Supporting Information 2: Testing Stationarity of State Attachment

| **Table S2**  *Testing Stationarity of State Attachment Dimensions: Percentages of Test that Rejected Stationarity in Sample I (N =122) and Sample II (N =127)* | | | | | | |
| --- | --- | --- | --- | --- | --- | --- |
|  | Sample I | | | Sample II | | |
| Stationarity Test | State Attachment Security | State  Attachment Avoidance | State Attachment Anxiety | State Attachment Security | State  Attachment Avoidance | State Attachment Anxiety |
| Kwiatkowski-Phillips-Schmidt-Shin test for a mean  H0: stationary mean  H1: Non-stationary mean | 19.7%  (*n* = 24) | 11.5%  (*n =* 14) | 14.8%  (*n* = 18) | 11.0%  (*n* = 14) | 23.6%  (*n =* 30) | 16.5%  (*n* = 21) |
|  |  |  |  |  |  |  |
| Kwiatkowski-Phillips-Schmidt-Shin test for a trend  H0: stationary trend  H1: nonstationary trend | 12.3%  (*n* = 15) | 3.3%  (*n* = 4) | 9.8%  (*n* = 12) | 9.4%  (*n* = 12) | 13.4%  (*n* = 17) | 14.2%  (*n* = 18) |
|  |  |  |  |  |  |  |
| *Notes.* These tests were conducted using the and tseries (Trapletti & Hornik, 2019) packages in R. The alpha level of .05/2 = .025 was used. This level was chosen as there were two tests per variable for each participant. | | | | | | |

## Supporting Information 3: Descriptive Statistics and Correlations of Study Variables

| **Table S3**  Descriptive Statistics and Correlations of Study Variables | | | | | | | | | | | | | |
| --- | --- | --- | --- | --- | --- | --- | --- | --- | --- | --- | --- | --- | --- |
| Descriptive Statistics: Sample I | | | | | | | | | | | | | |
|  | | *n* | | *M* | | *SD* | | Skewness | | Kurtosis | | ICC | |
| 1. State Attachment Security | | 4629 | | 5.687 | | 1.220 | | -0.672 | | -0.309 | | .706 | |
| 2. State Attachment Avoidance | | 4629 | | 2.376 | | 1.331 | | 0.894 | | 0.268 | | .555 | |
| 3. State Attachment Anxiety | | 4629 | | 4.200 | | 1.318 | | -0.025 | | -0.371 | | .484 | |
| 4. Trait Attachment Avoidance | | 122 | | 2.770 | | 0.906 | | 0.345 | | -0.683 | |  | |
| 5. Trait Attachment Anxiety | | 122 | | 3.255 | | 1.187 | | 0.069 | | -1.074 | |  | |
| 6. Time Spent Alone % | | 122 | | 0.375 | | 0.176 | | 0.571 | | -0.460 | |  | |
| 7. Romantic Relationship Status | | 122 | | 0.672 | | 0.471 | | -0.724 | | -1.487 | |  | |
| Descriptive Statistics: Sample II | | | | | | | | | | | | | |
|  | | *n* | | *M* | | *SD* | | Skewness | | Kurtosis | | ICC | |
| 1. State Attachment Security | | 5322 | | 5.955 | | 1.303 | | -1.481 | | 2.066 | | .758 | |
| 2. State Attachment Avoidance | | 5322 | | 2.390 | | 1.410 | | 0.840 | | -0.105 | | .673 | |
| 3. State Attachment Anxiety | | 5322 | | 3.235 | | 1.535 | | 0.421 | | -0.333 | | .697 | |
| 4. Trait Attachment Avoidance | | 127 | | 3.000 | | 1.042 | | 0.236 | | -0.579 | |  | |
| 5. Trait Attachment Anxiety | | 127 | | 3.185 | | 1.231 | | 0.503 | | -0.527 | |  | |
| 6. Time Spent Alone % | | 127 | | 0.125 | | 0.134 | | 1.432 | | 1.394 | |  | |
| 7. Romantic Relationship Status | | 127 | | 0.504 | | 0.502 | | -0.016 | | -2.015 | |  | |
| \| Correlations: Sample I (Below the Diagonal) and Sample II (Above the Diagonal) \| \| --- \| | | | | | | | | | | | | | |
| Within-Person Level | 1 | | 2 | | 3 | | 4 | |  | |  | |  |
| 1. State Attachment Security | – | | **-.237** | | **-.104** | | .000 | |  | |  | |  |
| 2. State Attachment Avoidance | **-.319** | | – | | **.054** | | -.025 | |  | |  | |  |
| 3. State Attachment Anxiety | **.082** | | **-.227** | | – | | -.020 | |  | |  | |  |
| 4. Time | **-.045** | | .011 | | **-.035** | | – | |  | |  | |  |
| Between-Person Level | 1 | | 2 | | 3 | | 4 | | 5 | | 6 | | 7 |
| 1. State Attachment Security | – | | **-.323** | | -.089 | | **-.411** | | **-.450** | | **-.387** | | **.391** |
| 2. State Attachment Avoidance | **-.538** | | – | | .073 | | **.471** | | **.234** | | .117 | | **-.296** |
| 3. State Attachment Anxiety | .025 | | -.090 | | – | | **-.201** | | **.327** | | **-.230** | | -.045 |
| 4. Trait Attachment Avoidance | **-.466** | | **.441** | | -.138 | | – | | **.276** | | **.303** | | **-.582** |
| 5. Trait Attachment Anxiety | **-.443** | | **.333** | | **.248** | | **.444** | | – | | .108 | | **-.427** |
| 6. Time Spent Alone % | **-.202** | | .160 | | -.170 | | .136 | | **.252** | | – | | -.161 |
| 7. Romantic Relationship Status | **.370** | | **-.212** | | -.033 | | **-.273** | | **-.321** | | **-.552** | | – |
| *Notes.* Within-person correlations are for group-mean-centered data, whereas correlations at the between-person level are for aggregated data. For bolded values, *p* < .050. *ICC* = Intraclass correlation. | | | | | | | | | | | | | |

## Supporting Information 4: Sensitivity Analyses for Associations of Dynamic Features Within Each State Attachment Dimension

**Figure S4A**

*Associations Between Dynamic Features of State Attachment Security (Inverted) and Avoidance and Their 95% Credible Intervals (CrIs) After Accounting Floor and Ceiling Effects with Two-Part Modeling*

*
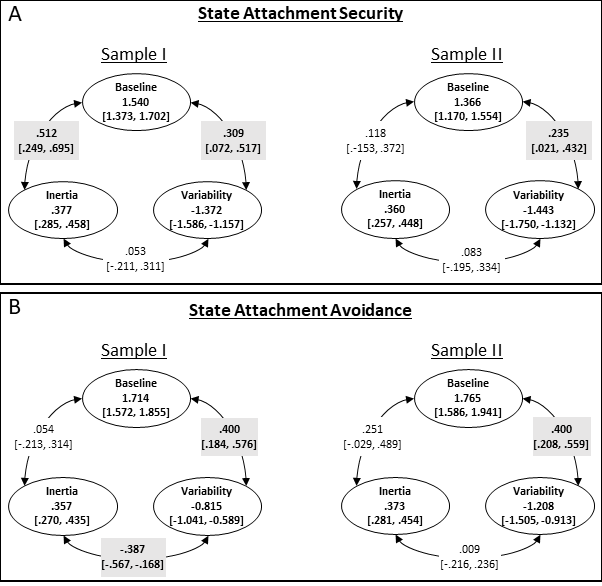
*

*Notes.* Before the analyses, both state attachment security and avoidance scales were transformed to vary from 0 to 6 instead of 1 to 7, as used in the primary analyses (see Figure 2). Moreover, the scale of state attachment security was reversed (0 = maximum amount of security and 6 = minimal amount of security) to allow two-part modeling.

**Figure S4B**

*Associations Between Dynamic Features of Each State Attachment Dimension and Their 95% Credible Intervals (CrIs) Using Previous Ecological Momentary Assessment Questionnaire as Lagged Observations*


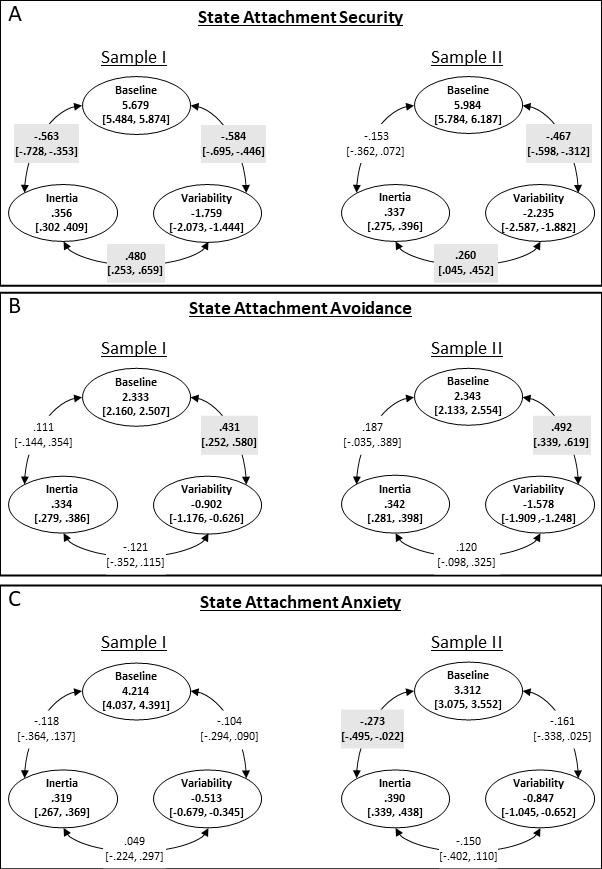


Notes. The values in the circles refer to the fixed estimates and their 95% credible intervals for baseline, variability, and inertia. Regarding correlations, the 95% CrIs do not include zero in bolded values surrounded by a grey area.

## Supporting Information 5: Unstandardized Associations of Trait Attachment with Dynamic Features of State Attachment

| **Table S5**  *Unstandardized Associations of Trait Attachment with Dynamic Features of State Attachment* | | | | | | |
| --- | --- | --- | --- | --- | --- | --- |
| **Model for State Attachment Security** |  | | | | | |
|  | Baseline  of State Attachment Security | | Variability  of State Attachment Security | | Inertia  of State Attachment Security | |
|  | Sample I | Sample II | Sample I | Sample II | Sample I | Sample II |
| Predictors | Posterior *Mdn*  β [95% CrI] | Posterior *Mdn*  β [95% CrI] | Posterior *Mdn*  β [95% CrI] | Posterior *Mdn*  β [95% CrI] | Posterior *Mdn*  β [95% CrI] | Posterior *Mdn*  β [95% CrI] |
| Trait Attachment Anxiety | **-0.207 [-0.364, -0.051]** | **-0.302 [-0.450, -0.156]** | **0.541 [0.266, 0.822]** | **0.426 [0.125, 0.724]** | 0.015 [-0.039, 0.069] | 0.015 [-0.045, 0.074] |
| Trait Attachment Avoidance | **-0.332 [-0.536, -0.131]** | -0.186 [-0.388, 0.014] | 0.059 [-0.300, 0.418] | **0.602 [0.197, 1.009]** | 0.069 [-0.001, 0.139] | 0.005 [-0.078, 0.086] |
| % Time Spent Alone | 0.070 [-1.018, 1.156] | **-2.309 [-3.591, -1.032]** | -1.308 [-3.293, 0.622] | 1.393 [-1.202, 3.968] | 0.067 [-0.323, 0.460] | -0.127 [-0.641, 0.384] |
| Romantic Relationship Status | **0.527 [0.101, 0.952]** | 0.235 [-0.191, 0.661] | -0.502 [-1.269, 0.253] | 0.760 [-0.107, 1.622] | 0.029 [-0.122, 0.178] | -0.006 [-0.176, 0.165] |
| *R*^2^ | .137 | .159 | .109 | .118 | .061 | .027 |
| **Model for State Attachment Avoidance** |  | | | | | |
|  | Baseline  of State Attachment Avoidance | | Variability  of State Attachment Avoidance | | Inertia  of State Attachment Avoidance | |
|  | Sample I | Sample II | Sample I | Sample II | Sample I | Sample II |
| Predictors | Posterior *Mdn*  β [95% CrI] | Posterior *Mdn*  β [95% CrI] | Posterior *Mdn*  β [95% CrI] | Posterior *Mdn*  β [95% CrI] | Posterior *Mdn*  β [95% CrI] | Posterior *Mdn*  β [95% CrI] |
| Trait Attachment Anxiety | 0.134 [-0.033, 0.302] | 0.100 [-0.072, 0.273] | 0.209 [-0.054, 0.476] | 0.136 [-0.159, 0.434] | -0.045 [-0.102, 0.011] | 0.003 [-0.050, 0.055] |
| Trait Attachment Avoidance | **0.409 [0.197, 0.620]** | **0.526 [0.292, 0.759]** | 0.069 [-0.276, 0.411] | **0.542 [0.135, 0.950]** | 0.016 [-0.057, 0.089] | 0.046 [-0.028, 0.120] |
| % Time Spent Alone | 0.246 [-0.930, 1.417] | -0.336 [-1.796, 1.133] | **-2.021 [-3.906, -0.172]** | -0.365 [-2.935, 2.210] | 0.252 [-0.151, 0.661] | -0.093 [-0.544, 0.357] |
| Romantic Relationship Status | -0.088 [-0.545, 0.368] | 0.035 [-0.451, 0.527] | -0.080 [-0.805, 0.636] | 0.614 [-0.237, 1.479] | 0.013 [-0.143, 0.169] | 0.013 [-0.142, 0.170] |
| *R*^2^ | .102 | .137 | .056 | .072 | .062 | .038 |
| **Model for State Attachment Anxiety** |  | | | | | |
|  | Baseline  of State Attachment Anxiety | | Variability  of State Attachment Anxiety | | Inertia  of State Attachment Anxiety | |
|  | Sample I | Sample II | Sample I | Sample II | Sample I | Sample II |
| Predictors | Posterior *Mdn*  β [95% CrI] | Posterior *Mdn*  β [95% CrI] | Posterior *Mdn*  β [95% CrI] | Posterior *Mdn*  β [95% CrI] | Posterior *Mdn*  β [95% CrI] | Posterior *Mdn*  β [95% CrI] |
| Trait Attachment Anxiety | **0.340 [0.186, 0.496]** | **0.425 [0.232, 0.618]** | 0.101 [-0.061, 0.263] | 0.173 [-0.012, 0.361] | -0.022 [-0.079, 0.033] | -0.007 [-0.055, 0.040] |
| Trait Attachment Avoidance | **-0.344 [-0.543, -0.146]** | **-0.377 [-0.641, -0.116]** | 0.042 [-0.166, 0.253] | 0.168 [-0.088, 0.425] | 0.003 [-0.067, 0.073] | 0.030 [-0.033, 0.093] |
| % Time Spent Alone | **-1.727 [-2.831, -0.641]** | **-1.875 [-3.541, -0.219]** | -0.605 [-1.759, 0.515] | -0.293 [-1.902, 1.307] | -0.023 [-0.419, 0.355] | 0.143 [-0.23, 0.517] |
| Romantic Relationship Status | -0.331 [-0.757, 0.090] | -0.203 [-0.762, 0.358] | 0.164 [-0.279, 0.601] | **0.775 [0.240, 1.317]** | -0.112 [-0.261, 0.032] | 0.088 [-0.041, 0.218] |
| *R*^2^ | .211 | .160 | .038 | .091 | .059 | .058 |
| *Notes. Notes.* In Sample I, *N_participants_* = 122, *N_observations_ =* 4629*.* In Sample II, *N_participants_* = 127, *N_observations_ =* 5322*.* In bolded values, the 95% credible interval (95% CrI) does not contain zero. The results were summarized in R using the MplusAutomation package (Hallquist & Wiley, 2018). | | | | | | |

## Supporting Information 6: Sensitivity Analyses for Associations of Trait Attachment with Dynamic Features of State Attachment

| **Table S6A**  *Unstandardized Associations of Trait Attachment with Dynamic Features of State Attachment After Covarying Emotions* | | | | | | |
| --- | --- | --- | --- | --- | --- | --- |
| **Model for State Attachment Security** |  | | | | | |
|  | Baseline  of State Attachment Security | | Variability  of State Attachment Security | | Inertia  of State Attachment Security | |
|  | Sample I | Sample II | Sample I | Sample II | Sample I | Sample II |
| Predictors | Posterior *Mdn*  β [95% CrI] | Posterior *Mdn*  β [95% CrI] | Posterior *Mdn*  β [95% CrI] | Posterior *Mdn*  β [95% CrI] | Posterior *Mdn*  β [95% CrI] | Posterior *Mdn*  β [95% CrI] |
| Trait Attachment Anxiety | **-0.151 [-0.301, -0.001]** | -0.105 [-0.253, 0.043] | **0.448 [0.141, 0.757]** | 0.213 [-0.111, 0.541] | 0.015 [-0.045, 0.073] | -0.020 [-0.088, 0.046] |
| Trait Attachment Avoidance | **-0.196 [-0.383, -0.009]** | -0.044 [-0.230, 0.141] | 0.030 [-0.355, 0.408] | 0.409 [-0.007, 0.816] | 0.044 [-0.030, 0.118] | -0.012 [-0.098, 0.073] |
| % Time Spent Alone | 0.271 [-0.688, 1.235] | -**1.975 [-3.151, -0.814]** | -1.431 [-3.387, 0.521] | 0.769 [-1.795, 3.367] | 0.036 [-0.358, 0.430] | -0.109 [-0.635, 0.403] |
| Romantic Relationship Status | **0.628 [0.255, 1.005]** | **0.555 [0.161, 0.951]** | -0.537 [-1.304, 0.230] | 0.358 [-0.517, 1.229] | 0.013 [-0.136, 0.163] | -0.042 [-0.219, 0.136] |
| Average EMA Negative Emotions | -0.446 [-1.007, 0.119] | **-0.024 [-0.035, -0.012]** | 0.772 [-0.377, 1.906] | 0.019 [-0.007, 0.045] | -0.008 [-0.235, 0.215] | 0.005 [-0.001, 0.010] |
| Average EMA Positive Emotions | **0.822 [0.518, 1.130]** | **0.020 [0.010, 0.030]** | -0.329 [-0.954, 0.292] | **-0.030 [-0.052, -0.008]** | **-0.130 [-0.255, -0.007]** | -0.002 [-0.006, 0.002] |
| *R*^2^ | .244 | .241 | .113 | .109 | .092 | .068 |
| **Model for State Attachment Avoidance** |  | | | | | |
|  | Baseline  of State Attachment Avoidance | | Variability  of State Attachment Avoidance | | Inertia  of State Attachment Avoidance | |
|  | Sample I | Sample II | Sample I | Sample II | Sample I | Sample II |
| Predictors | Posterior *Mdn*  β [95% CrI] | Posterior *Mdn*  β [95% CrI] | Posterior *Mdn*  β [95% CrI] | Posterior *Mdn*  β [95% CrI] | Posterior *Mdn*  β [95% CrI] | Posterior *Mdn*  β [95% CrI] |
| Trait Attachment Anxiety | 0.061 [-0.113, 0.236] | -0.044 [-0.229, 0.142] | 0.082 [-0.208, 0.369] | -0.072 [-0.398, 0.249] | -0.033 [-0.095, 0.030] | -0.003 [-0.065, 0.057] |
| Trait Attachment Avoidance | **0.337 [0.122, 0.553]** | **0.432 [0.197, 0.665]** | 0.043 [-0.317, 0.399] | 0.361 [-0.052, 0.768] | 0.011 [-0.069, 0.090] | 0.048 [-0.032, 0.127] |
| % Time Spent Alone | 0.089 [-1.026, 1.199] | -0.432 [-1.873, 1.006] | **-2.178 [-4.024, -0.318]** | -0.930 [-3.472, 1.653] | 0.268 [-0.134, 0.673] | -0.069 [-0.535, 0.401] |
| Romantic Relationship Status | -0.146 [-0.581, 0.288] | -0.184 [-0.669, 0.306] | -0.111 [-0.83, 0.601] | 0.229 [-0.636, 1.105] | 0.014 [-0.141, 0.172] | 0.011 [-0.156, 0.180] |
| Average EMA Negative Emotions | 0.595 [-0.051, 1.243] | **0.022 [0.008, 0.037]** | **1.075 [0.006, 2.155]** | 0.020 [-0.005, 0.046] | -0.134 [-0.39, 0.119] | 0.002 [-0.003, 0.006] |
| Average EMA Positive Emotions | **-0.508 [-0.867, -0.153]** | -0.01 [-0.022, 0.002] | -0.345 [-0.929, 0.241] | **-0.028 [-0.049, -0.006]** | -0.018 [-0.141, 0.103] | 0.000 [-0.004, 0.004] |
| *R*^2^ | .136 | .164 | .087 | .093 | .083 | .058 |
| **Model for State Attachment Anxiety** |  | | | | | |
|  | Baseline  of State Attachment Anxiety | | Variability  of State Attachment Anxiety | | Inertia  of State Attachment Anxiety | |
|  | Sample I | Sample II | Sample I | Sample II | Sample I | Sample II |
| Predictors | Posterior *Mdn*  β [95% CrI] | Posterior *Mdn*  β [95% CrI] | Posterior *Mdn*  β [95% CrI] | Posterior *Mdn*  β [95% CrI] | Posterior *Mdn*  β [95% CrI] | Posterior *Mdn*  β [95% CrI] |
| Trait Attachment Anxiety | **0.275 [0.111, 0.439]** | **0.261 [0.055, 0.468]** | 0.046 [-0.133, 0.228] | 0.097 [-0.110, 0.300] | -0.015 [-0.078, 0.047] | 0.003 [-0.051, 0.056] |
| Trait Attachment Avoidance | **-0.242 [-0.446, -0.037]** | **-0.425 [-0.687, -0.167]** | 0.092 [-0.133, 0.313] | 0.060 [-0.199, 0.317] | -0.014 [-0.092, 0.063] | 0.031 [-0.035, 0.097] |
| % Time Spent Alone | **-1.649 [-2.723, -0.587]** | **-1.676 [-3.309, -0.059]** | -0.599 [-1.744, 0.544] | -0.775 [-2.373, 0.832] | -0.049 [-0.461, 0.345] | 0.120 [-0.259, 0.505] |
| Romantic Relationship Status | -0.263 [-0.674, 0.151] | -0.365 [-0.917, 0.180] | 0.19 [-0.258, 0.636] | **0.581 [0.032, 1.129]** | -0.125 [-0.277, 0.022] | 0.095 [-0.042, 0.233] |
| Average Daily Negative Emotions | 0.584 [-0.029, 1.197] | **0.032 [0.015, 0.048]** | 0.473 [-0.195, 1.133] | 0.000 [-0.016, 0.017] | -0.062 [-0.296, 0.167] | -0.003 [-0.007, 0.001] |
| Average Daily Positive Emotions | **0.467 [0.135, 0.803]** | -0.001 [-0.015, 0.013] | 0.173 [-0.194, 0.536] | **-0.019 [-0.033, -0.006]** | -0.080 [-0.223, 0.055] | 0.000 [-0.004, 0.003] |
| *R*^2^ | .213 | .199 | .062 | .107 | .096 | .091 |
| *Notes. Notes.* In Sample I, *N_participants_* = 122, *N_observations_ =* 4629*.* In Sample II, *N_participants_* = 127, *N_observations_ =* 5322*.* In bolded values, the 95% credible interval (95% CrI) does not contain zero. The results were summarized in R using the MplusAutomation package (Hallquist & Wiley, 2018). | | | | | | |

| **Table S6B**  *Standardized Associations of Trait Attachment with Dynamic Features of State Attachment After Covarying Emotions* | | | | | | |
| --- | --- | --- | --- | --- | --- | --- |
| **Model for State Attachment Security** |  | | | | | |
|  | Baseline  of State Attachment Security | | Variability  of State Attachment Security | | Inertia  of State Attachment Security | |
|  | Sample I | Sample II | Sample I | Sample II | Sample I | Sample II |
| Predictors | Posterior *Mdn*  β [95% CrI] | Posterior *Mdn*  β [95% CrI] | Posterior *Mdn*  β [95% CrI] | Posterior *Mdn*  β [95% CrI] | Posterior *Mdn*  β [95% CrI] | Posterior *Mdn*  β [95% CrI] |
| Trait Attachment Anxiety | **-0.145 [-0.284, -0.001]** | -0.098 [-0.236, 0.038] | **0.225 [0.071, 0.367]** | 0.097 [-0.050, 0.239] | 0.050 [-0.141, 0.235] | -0.055 [-0.224, 0.123] |
| Trait Attachment Avoidance | **-0.144 [-0.277, -0.006]** | -0.035 [-0.183, 0.106] | 0.011 [-0.130, 0.155] | 0.157 [-0.003, 0.303] | 0.110 [-0.074, 0.287] | -0.028 [-0.214, 0.163] |
| % Time Spent Alone | 0.039 [-0.098, 0.168] | **-0.202 [-0.316, -0.082]** | -0.107 [-0.242, 0.039] | 0.038 [-0.087, 0.166] | 0.017 [-0.170, 0.201] | -0.032 [-0.182, 0.118] |
| Romantic Relationship Status | **0.240 [0.097, 0.369]** | **0.211 [0.062, 0.346]** | -0.107 [-0.251, 0.045] | 0.066 [-0.096, 0.219] | 0.017 [-0.172, 0.204] | -0.046 [-0.23, 0.147] |
| Average EMA Negative Emotions | -0.104 [-0.233, 0.027] | **-0.244 [-0.355, -0.122]** | 0.094 [-0.045, 0.230] | 0.094 [-0.036, 0.222] | -0.007 [-0.183, 0.165] | 0.140 [-0.015, 0.285] |
| Average EMA Positive Emotions | **0.330 [0.209, 0.437]** | **0.252 [0.126, 0.367]** | -0.069 [-0.195, 0.061] | **-0.185 [-0.315, -0.047]** | **-0.178 [-0.341, -0.009]** | -0.072 [-0.225, 0.087] |
| *R*^2^ | .244 | .241 | .113 | .109 | .092 | .068 |
| **Model for State Attachment Avoidance** |  | | | | | |
|  | Baseline  of State Attachment Avoidance | | Variability  of State Attachment Avoidance | | Inertia  of State Attachment Avoidance | |
|  | Sample I | Sample II | Sample I | Sample II | Sample I | Sample II |
| Predictors | Posterior *Mdn*  β [95% CrI] | Posterior *Mdn*  β [95% CrI] | Posterior *Mdn*  β [95% CrI] | Posterior *Mdn*  β [95% CrI] | Posterior *Mdn*  β [95% CrI] | Posterior *Mdn*  β [95% CrI] |
| Trait Attachment Anxiety | 0.054 [-0.096, 0.208] | -0.034 [-0.175, 0.112] | 0.045 [-0.111, 0.196] | -0.033 [-0.178, 0.114] | -0.104 [-0.290, 0.091] | -0.010 [-0.188, 0.171] |
| Trait Attachment Avoidance | **0.226 [0.081, 0.360]** | **0.288 [0.131, 0.427]** | 0.018 [-0.129, 0.162] | 0.140 [-0.019, 0.289] | 0.026 [-0.163, 0.208] | 0.122 [-0.079, 0.309] |
| % Time Spent Alone | 0.012 [-0.131, 0.153] | -0.037 [-0.158, 0.086] | **-0.175 [-0.311, -0.026]** | -0.047 [-0.17, 0.082] | 0.125 [-0.061, 0.303] | -0.023 [-0.174, 0.129] |
| Romantic Relationship Status | -0.051 [-0.198, 0.098] | -0.059 [-0.213, 0.095] | -0.024 [-0.173, 0.129] | 0.043 [-0.118, 0.201] | 0.017 [-0.174, 0.206] | 0.014 [-0.188, 0.213] |
| Average EMA Negative Emotions | 0.127 [-0.011, 0.259] | **0.197 [0.066, 0.317]** | **0.142 [0.001, 0.277]** | 0.105 [-0.026, 0.231] | -0.102 [-0.289, 0.090] | 0.054 [-0.101, 0.205] |
| Average EMA Positive Emotions | **-0.187 [-0.313, -0.055]** | -0.106 [-0.234, 0.027] | -0.078 [-0.206, 0.054] | **-0.175 [-0.303, -0.038]** | -0.024 [-0.181, 0.133] | 0.020 [-0.143, 0.180] |
| *R*^2^ | .136 | .164 | .087 | .093 | .083 | .058 |
| **Model for State Attachment Anxiety** |  | | | | | |
|  | Baseline  of State Attachment Anxiety | | Variability  of State Attachment Anxiety | | Inertia  of State Attachment Anxiety | |
|  | Sample I | Sample II | Sample I | Sample II | Sample I | Sample II |
| Predictors | Posterior *Mdn*  β [95% CrI] | Posterior *Mdn*  β [95% CrI] | Posterior *Mdn*  β [95% CrI] | Posterior *Mdn*  β [95% CrI] | Posterior *Mdn*  β [95% CrI] | Posterior *Mdn*  β [95% CrI] |
| Trait Attachment Anxiety | **0.246 [0.099, 0.377]** | **0.179 [0.037, 0.315]** | 0.042 [-0.118, 0.203] | 0.072 [-0.080, 0.215] | -0.049 [-0.254, 0.155] | 0.013 [-0.192, 0.207] |
| Trait Attachment Avoidance | **-0.164 [-0.290, -0.025]** | **-0.246 [-0.379, -0.097]** | 0.063 [-0.091, 0.209] | 0.038 [-0.124, 0.191] | -0.036 [-0.228, 0.159] | 0.102 [-0.113, 0.298] |
| % Time Spent Alone | **-0.218 [-0.343, -0.078]** | **-0.126 [-0.244, -0.004]** | -0.081 [-0.230, 0.072] | -0.063 [-0.188, 0.067] | -0.025 [-0.212, 0.174] | 0.050 [-0.108, 0.207] |
| Romantic Relationship Status | -0.093 [-0.230, 0.053] | -0.102 [-0.244, 0.050] | 0.069 [-0.092, 0.223] | **0.176 [0.010, 0.327]** | -0.168 [-0.345, 0.029] | 0.149 [-0.064, 0.341] |
| Average EMA Negative Emotions | 0.127 [-0.006, 0.257] | **0.240 [0.115, 0.357]** | 0.104 [-0.043, 0.243] | 0.002 [-0.128, 0.134] | -0.051 [-0.237, 0.133] | -0.114 [-0.282, 0.059] |
| Average EMA Positive Emotions | **0.174 [0.050, 0.294]** | -0.008 [-0.133, 0.122] | 0.066 [-0.073, 0.198] | **-0.196 [-0.327, -0.056]** | -0.112 [-0.291, 0.077] | -0.013 [-0.188, 0.161] |
| *R*^2^ | .213 | .199 | .062 | .107 | .096 | .091 |
| *Notes. Notes.* In Sample I, *N_participants_* = 122, *N_observations_ =* 4629*.* In Sample II, *N_participants_* = 127, *N_observations_ =* 5322*.* In bolded values, the 95% credible interval (95% CrI) does not contain zero. The results were summarized in R using the MplusAutomation package (Hallquist & Wiley, 2018). | | | | | | |

| **Table S6C**  *Unstandardized Associations of Trait Attachment with Dynamic Features of State Attachment Using Previous Ecological Momentary Assessment Questionnaire as Lagged Observations* | | | | | | |
| --- | --- | --- | --- | --- | --- | --- |
| **Model for State Attachment Security** |  | | | | | |
|  | Baseline  of State Attachment Security | | Variability  of State Attachment Security | | Inertia  of State Attachment Security | |
|  | Sample I | Sample II | Sample I | Sample II | Sample I | Sample II |
| Predictors | Posterior *Mdn*  β [95% CrI] | Posterior *Mdn*  β [95% CrI] | Posterior *Mdn*  β [95% CrI] | Posterior *Mdn*  β [95% CrI] | Posterior *Mdn*  β [95% CrI] | Posterior *Mdn*  β [95% CrI] |
| Trait Attachment Anxiety | **-0.213 [-0.370, -0.056]** | **-0.297 [-0.445, -0.149]** | **0.541 [0.261, 0.823]** | **0.424 [0.125, 0.716]** | 0.005 [-0.045, 0.055] | 0.024 [-0.031, 0.078] |
| Trait Attachment Avoidance | **-0.349 [-0.554, -0.144]** | -0.185 [-0.385, 0.014] | 0.083 [-0.288, 0.447] | **0.614 [0.212, 1.015]** | **0.077 [0.012, 0.142]** | -0.009 [-0.085, 0.066] |
| % Time Spent Alone | 0.080 [-1.027, 1.200] | **-2.321 [-3.591, -1.053]** | -1.310 [-3.326, 0.687] | 1.333 [-1.228, 3.889] | 0.060 [-0.305, 0.425] | -0.047 [-0.513, 0.426] |
| Romantic Relationship Status | **0.494 [0.063, 0.927]** | 0.231 [-0.191, 0.656] | -0.444 [-1.218, 0.322] | 0.763 [-0.102, 1.628] | -0.020 [-0.156, 0.113] | 0.001 [-0.158, 0.159] |
| *R*^2^ | .136 | .158 | .103 | .120 | .075 | .030 |
| **Model for State Attachment Avoidance** |  | | | | | |
|  | Baseline  of State Attachment Avoidance | | Variability  of State Attachment Avoidance | | Inertia  of State Attachment Avoidance | |
|  | Sample I | Sample II | Sample I | Sample II | Sample I | Sample II |
| Predictors | Posterior *Mdn*  β [95% CrI] | Posterior *Mdn*  β [95% CrI] | Posterior *Mdn*  β [95% CrI] | Posterior *Mdn*  β [95% CrI] | Posterior *Mdn*  β [95% CrI] | Posterior *Mdn*  β [95% CrI] |
| Trait Attachment Anxiety | 0.116 [-0.036, 0.268] | 0.102 [-0.072, 0.272] | 0.187 [-0.078, 0.454] | 0.151 [-0.143, 0.443] | -0.034 [-0.085, 0.016] | -0.004 [-0.057, 0.048] |
| Trait Attachment Avoidance | **0.335 [0.140, 0.531]** | **0.529 [0.297, 0.759]** | 0.070 [-0.270, 0.409] | **0.563 [0.165, 0.959]** | 0.028 [-0.038, 0.094] | 0.036 [-0.037, 0.108] |
| % Time Spent Alone | 0.271 [-0.796, 1.340] | -0.311 [-1.763, 1.156] | -1.883 [-3.762, 0.008] | -0.409 [-2.946, 2.121] | 0.182 [-0.182, 0.541] | -0.149 [-0.596, 0.305] |
| Romantic Relationship Status | -0.203 [-0.617, 0.213] | 0.042 [-0.450, 0.530] | -0.060 [-0.780, 0.665] | 0.625 [-0.233, 1.481] | 0.022 [-0.117, 0.161] | -0.006 [-0.160, 0.147] |
| *R*^2^ | .096 | .139 | .050 | .076 | .056 | .034 |
| **Model for State Attachment Anxiety** |  | | | | | |
|  | Baseline  of State Attachment Anxiety | | Variability  of State Attachment Anxiety | | Inertia  of State Attachment Anxiety | |
|  | Sample I | Sample II | Sample I | Sample II | Sample I | Sample II |
| Predictors | Posterior *Mdn*  β [95% CrI] | Posterior *Mdn*  β [95% CrI] | Posterior *Mdn*  β [95% CrI] | Posterior *Mdn*  β [95% CrI] | Posterior *Mdn*  β [95% CrI] | Posterior *Mdn*  β [95% CrI] |
| Trait Attachment Anxiety | **0.342 [0.186, 0.495]** | **0.427 [0.233, 0.620]** | 0.089 [-0.073, 0.251] | **0.181 [0.008, 0.355]** | -0.027 [-0.074, 0.022] | -0.030 [-0.077, 0.017] |
| Trait Attachment Avoidance | **-0.345 [-0.544, -0.145]** | **-0.378 [-0.641, -0.117]** | 0.053 [-0.158, 0.262] | 0.169 [-0.066, 0.403] | -0.013 [-0.077, 0.049] | 0.027 [-0.035, 0.090] |
| % Time Spent Alone | **-1.731 [-2.821, -0.635]** | **-1.868 [-3.532, -0.204]** | -0.621 [-1.769, 0.528] | -0.283 [-1.761, 1.205] | 0.119 [-0.217, 0.457] | 0.169 [-0.201, 0.542] |
| Romantic Relationship Status | -0.331 [-0.757, 0.092] | -0.195 [-0.750, 0.362] | 0.110 [-0.329, 0.554] | **0.781 [0.275, 1.288]** | -0.075 [-0.202, 0.054] | 0.064 [-0.066, 0.193] |
| *R*^2^ | .211 | .161 | .035 | .105 | .061 | .066 |
| *Notes. Notes.* In Sample I, *N_participants_* = 122, *N_observations_ =* 4629*.* In Sample II, *N_participants_* = 127, *N_observations_ =* 5322*.* In bolded values, the 95% credible interval (95% CrI) does not contain zero. The results were summarized in R using the MplusAutomation package (Hallquist & Wiley, 2018). | | | | | | |

| **Table S6D**  *Standardized Associations of Trait Attachment with Dynamic Features of State Attachment Using Previous Ecological Momentary Assessment Questionnaire as Lagged Observations* | | | | | | |
| --- | --- | --- | --- | --- | --- | --- |
| **Model for State Attachment Security** | | | | | | |
|  | Baseline  of State Attachment Security | | Variability  of State Attachment Security | | Inertia  of State Attachment Security | |
|  | Sample I | Sample II | Sample I | Sample II | Sample I | Sample II |
| Predictors | Posterior *Mdn*  β* [95% CrI] | Posterior *Mdn*  β* [95% CrI] | Posterior *Mdn*  β* [95% CrI] | Posterior *Mdn*  β* [95% CrI] | Posterior *Mdn*  β* [95% CrI] | Posterior *Mdn*  β* [95% CrI] |
| Trait Attachment Anxiety | **-0.187 [-0.319, -0.048]** | **-0.259 [-0.378, -0.130]** | **0.267 [0.129, 0.391]** | **0.185 [0.055, 0.304]** | 0.019 [-0.156, 0.197] | 0.070 [-0.092, 0.227] |
| Trait Attachment Avoidance | **-0.233 [-0.361, -0.094]** | -0.137 [-0.279, 0.010] | 0.031 [-0.104, 0.166] | **0.228 [0.079, 0.358]** | **0.212 [0.031, 0.376]** | -0.023 [-0.207, 0.162] |
| % Time Spent Alone | 0.010 [-0.133, 0.150] | **-0.222 [-0.336, -0.100]** | -0.096 [-0.232, 0.050] | 0.064 [-0.058, 0.185] | 0.033 [-0.158, 0.219] | -0.015 [-0.163, 0.135] |
| Romantic Relationship Status | **0.172 [0.021, 0.314]** | 0.082 [-0.066, 0.232] | -0.087 [-0.230, 0.062] | 0.137 [-0.019, 0.274] | -0.028 [-0.218, 0.157] | 0.002 [-0.187, 0.186] |
| *R*^2^ | .136 | .158 | .103 | .120 | .075 | .030 |
| **Model for State Attachment Avoidance** |  | | | | | |
|  | Baseline  of State Attachment Avoidance | | Variability  of State Attachment Avoidance | | Inertia  of State Attachment Avoidance | |
|  | Sample I | Sample II | Sample I | Sample II | Sample I | Sample II |
| Predictors | Posterior *Mdn*  β* [95% CrI] | Posterior *Mdn*  β* [95% CrI] | Posterior *Mdn*  β* [95% CrI] | Posterior *Mdn*  β* [95% CrI] | Posterior *Mdn*  β* [95% CrI] | Posterior *Mdn*  β* [95% CrI] |
| Trait Attachment Anxiety | 0.110 [-0.033, 0.250] | 0.078 [-0.055, 0.205] | 0.102 [-0.042, 0.240] | 0.068 [-0.064, 0.195] | -0.123 [-0.292, 0.058] | -0.012 [-0.169, 0.146] |
| Trait Attachment Avoidance | **0.241 [0.099, 0.371]** | **0.345 [0.197, 0.471]** | 0.029 [-0.109, 0.166] | **0.216 [0.064, 0.350]** | 0.077 [-0.102, 0.247] | 0.093 [-0.094, 0.270] |
| % Time Spent Alone | 0.038 [-0.109, 0.183] | -0.026 [-0.145, 0.098] | -0.152 [-0.291, 0.001] | -0.020 [-0.142, 0.104] | 0.096 [-0.094, 0.276] | -0.050 [-0.198, 0.100] |
| Romantic Relationship Status | -0.076 [-0.227, 0.078] | 0.013 [-0.142, 0.159] | -0.013 [-0.163, 0.141] | 0.116 [-0.044, 0.259] | 0.032 [-0.162, 0.221] | -0.008 [-0.197, 0.178] |
| *R*^2^ | .096 | .139 | .050 | .076 | .056 | .034 |
| **Model for State Attachment Anxiety** |  | | | | | |
|  | Baseline  of State Attachment Anxiety | | Variability  of State Attachment Anxiety | | Inertia  of State Attachment Anxiety | |
|  | Sample I | Sample II | Sample I | Sample II | Sample I | Sample II |
| Predictors | Posterior *Mdn*  β* [95% CrI] | Posterior *Mdn*  β* [95% CrI] | Posterior *Mdn*  β* [95% CrI] | Posterior *Mdn*  β* [95% CrI] | Posterior *Mdn*  β* [95% CrI] | Posterior *Mdn*  β* [95% CrI] |
| Trait Attachment Anxiety | **0.294 [0.162, 0.408]** | **0.284 [0.155, 0.398]** | 0.081 [-0.066, 0.223] | **0.140 [0.006, 0.263]** | -0.105 [-0.283, 0.083] | -0.119 [-0.298, 0.065] |
| Trait Attachment Avoidance | **-0.225 [-0.340, -0.096]** | **-0.213 [-0.346, -0.066]** | 0.037 [-0.107, 0.179] | 0.110 [-0.043, 0.251] | -0.040 [-0.225, 0.142] | 0.090 [-0.119, 0.286] |
| % Time Spent Alone | **-0.220 [-0.344, -0.082]** | **-0.136 [-0.253, -0.015]** | -0.084 [-0.233, 0.070] | -0.024 [-0.146, 0.101] | 0.069 [-0.121, 0.261] | 0.073 [-0.086, 0.230] |
| Romantic Relationship Status | -0.113 [-0.248, 0.032] | -0.053 [-0.197, 0.097] | 0.040 [-0.117, 0.196] | **0.247 [0.089, 0.383]** | -0.117 [-0.307, 0.082] | 0.104 [-0.105, 0.298] |
| *R*^2^ | .211 | .161 | .035 | .105 | .061 | .066 |
| *Notes. Notes.* In Sample I, *N_participants_* = 122, *N_observations_ =* 4629*.* In Sample II, *N_participants_* = 127, *N_observations_ =* 5322*.* In bolded values, the 95% credible interval (95% CrI) does not contain zero. The results were summarized in R using the MplusAutomation package (Hallquist & Wiley, 2018). | | | | | | |

## References

Brosseau-Liard, P. E., & Savalei, V. (2014). Adjusting incremental fit indices for nonnormality. *Multivariate Behavioral Research*, *49*(5), 460–470. <https://doi.org/10.1080/00273171.2014.933697>

Brosseau-Liard, P. E., Savalei, V., & Li, L. (2012). An investigation of the sample performance of two nonnormality corrections for RMSEA. *Multivariate Behavioral Research*, *47*(6), 904–930. <https://doi.org/10.1080/00273171.2012.715252>

Burnham, K. P., & Anderson, D. R. (2004). Multimodel inference: understanding AIC and BIC in model selection. *Sociological Methods & Research, 33*(2), 261–304. doi:10.1177/0049124104268644

Hallquist, M. N., & Wiley, J. F. (2018). Mplus Automation: An R package for facilitating large-scale latent variable analyses in Mplus. *Structural Equation Modeling*, *25*(4), 621–638. https://doi.org/10.1080/10705511.2017.1402334

Hu, L.-t., & Bentler, P. M. (1999). Cutoff criteria for fit indexes in covariance structure analysis: Conventional criteria versus new alternatives. *Structural Equation Modelling, 6*(1), 1–55. https://psycnet.apa.org/doi/10.1080/10705519909540118

Jak, S. (2019). Cross-level invariance in multilevel factor models. *Structural Equation Modelling, 26*(4), 607–622. [https://doi.org/10.1080/10705511.2018.1534205](https://psycnet.apa.org/doi/10.1080/10705511.2018.1534205)

Jak, S., & Jorgensen, T. D. (2017). Relating measurement invariance, cross-level invariance, and multilevel reliability. *Frontiers in Psychology*, *8*, 1640. https://doi.org/10.3389/fpsyg.2017.01640

Sadikaj, G., Wright, A.G.C., Dunkley, D., Zuroff, D. & Moskowitz, D.S. (2021). Multilevel structural equation modelling for intensive longitudinal data: A practical guide for personality researchers. In J.F. Rauthmann (Ed.) *Handbook of Personality Dynamics* *and Processes* (pp. 856–887). Elsevier. <https://doi.org/10.1016/B978-0-12-813995-0.00033-9>

Satorra, A., & Bentler, P. M. (2001). A scaled difference chi-square test statistic for moment structure analysis. *Psychometrika, 66*(4), 507–514. <https://doi.org/10.1007/BF02296192>

Tammilehto, J., Bosmans, G., Kuppens, P., Flykt, M., Peltonen, K., Kerns, K., & Lindblom, J. (2022). Dynamics of attachment and emotion regulation in daily life: Uni- and bidirectional associations. *Cognition & Emotion, 36*(6), 1109–1131. <https://doi.org/10.1080/02699931.2022.2081534>

Trapletti, A. & Hornik, K. (2019). tseries: Time series analysis and computational finance. R package version 0.10-47. <https://CRAN.R-project.org/package=tseries>
